# TFE3 and TP53 were novel diagnostic biomarkers related to mitochondrial autophagy in chronic rhinosinusitis with nasal polyps

**DOI:** 10.3389/fgene.2024.1423778

**Published:** 2024-10-08

**Authors:** Ning Wang, Ying Yuan, Yanjun Jia, Yue Han, Xuemin Yu, Ying Fu, Xiao Li

**Affiliations:** ^1^ Department of Otolaryngology-Head and Neck Surgery, Qilu Hospital of Shandong University, Qingdao, China; ^2^ Department of Otolaryngology-Head and Neck Surgery, Qingdao Central Hospital, University of Health and Rehabilitation Sciences, Qingdao, China

**Keywords:** chronic rhinosinusitis with nasal polyps, mitochondrial autophagy, biomarkers, TFE3, TP53

## Abstract

**Background:**

Chronic rhinosinusitis with nasal polyps (CRSwNP) belongs to a subtype of Chronic rhinosinusitis which is a heterogeneous inflammatory condition. It has been reported that mitophagy may provide a new therapeutic option for CRSwNP.

**Methods:**

The GSE136825 (training dataset) and GSE179265 (validation dataset) were scoured from the Gene Expression Omnibus database. The candidate genes related to mitophagy were identified by differential expression analysis. Subsequently, the biomarkers were selected from the machine learning, Receiver Operating Characteristic curves, and expression level verification. A backpropagation (BP) neural network was generated to evaluate the diagnostic ability of biomarkers. In addition, the infiltration abundance of immune cells, potential drugs, and related ear-nose-throat (ENT) diseases were analyzed based on the biomarkers. Finally, qPCR analysis was performed to verify these biomarkers.

**Results:**

A total of 8 candidate genes were identified by overlapping 3,400 differentially expressed genes (DEGs) and 72 mitophagy-related genes Subsequently, TFE3 and TP53 were identified as biomarkers of CRSwNP, and the area under the curves (AUC) of the BP neural network was 0.74, which indicated that the biomarkers had excellent abilities. TFE3 and TP53 were co-enriched in the cancer pathway, cell cycle, endocytosis, etc. What’s more, Macrophage and Immature dendritic cells had significant correlations with biomarkers. The drugs (Doxorubicin, Tetrachlorodibenzodioxin, etc.) and the ear-nose-throat diseases (hearing loss, sensorineural, tinnitus, etc.) related to biomarkers were predicted. Ultimately, qPCR results showed that the expression levels of TFE3 and TP53 in polyp tissue of CRSwNP were increased.

**Conclusion:**

Overall, TFE3 and TP53 could be used as biomarkers or potential therapeutic targets to diagnose and treat CRSwNP.

## 1 Introduction

Chronic rhinosinusitis (CRS) is a common disease, affecting 5% of the general population (Fokkens et al., 2020). It is a chronic inflammation of the nasal cavity and sinuses, characterized by symptoms such as nasal congestion, rhinorrhea, facial pressure, and loss of smell. Chronic rhinosinusitis with nasal polyps (CRSwNP) is a severe form of CRS, accounting for 18% of CRS patients (Benjamin et al., 2019). CRSwNP is closely associated with adult asthma, decreased health-related quality of life (HRQoL) (Bachert et al., 2015; Khan et al., 2019), and economic burden (Luke, 2017). The pathogenesis of CRSwNP is generally believed to be the result of interactions among various factors, including pathogenic microorganisms, genetic factors, and immune mechanisms. Regarding the high risk of recurrence after endoscopic sinus surgery for CRSwNP, previous studies have revealed that certain biomarkers such as c-Src, SMAD3, and CDH1 are associated with this phenomenon (Lee et al., 2017; Byoungjae et al., 2018; Jung et al., 2018). Macrophages in CRSwNP exhibit dysregulation of pro-inflammatory mediators, decreased phagocytosis, and impaired autophagy, which not only hinder the effective resolution of inflammation but also promote pathological processes such as fibrotic deposition, increased vascular permeability, and extracellular matrix degradation, thereby exacerbating disease progression and polyp formation (Fan et al., 2024). Therefore, a comprehensive understanding of the complex relationship between inflammation and chronic rhinosinusitis with nasal polyps (CRSwNP) is crucial for the development of more effective therapeutic strategies. Given the heterogeneity of chronic rhinosinusitis with nasal polyps (CRSwNP) and the complex cellular and molecular pathophysiological mechanisms involved, the identification of subtype-based biomarkers is crucial for the development of personalized treatment strategies. Currently, although several biomarkers associated with eosinophilic chronic rhinosinusitis, nasal polyps, and disease progression have been identified, the subtypes of chronic rhinosinusitis remain incompletely characterized, and the combinations of biomarkers that can accurately reflect these subtypes are yet to be fully explored. With the advancement of machine learning technologies, the trend is shifting towards utilizing multiple integrated biomarkers rather than relying on a single indicator to predict disease progression and treatment outcomes. This approach not only aids in deepening the understanding of CRSwNP but also provides robust support for enhancing treatment precision and improving patient prognosis (Nakayama and Haruna, 2023). Therefore, it is necessary to investigate more indicative biomarkers in order to provide new insights for the development of treatment strategies for CRSwNP.

Mitophagy refers to the selective wrapping and degradation of damaged mitochondria by cellular autophagy mechanisms, which is an important regulatory mechanism that ensures the balance of mitochondrial quantity and quality, thereby maintaining cellular survival (Vives-Bauza et al., 2010). Mitophagy is a process in which mitochondria depolarize under pathological conditions, leading to mitochondrial damage. Subsequently, damaged mitochondria are recognized by autophagosomes and undergo fusion with lysosomes to complete degradation (Wu et al., 2017). Mitophagy can be mediated by various receptors, including the Par-kin/PINK1 pathway-mediated mitophagy and B-cell leukemia/lymphoma 2 (BCL2)/adenovirus E1B19kD-interacting protein 3 (BNIP3), BNIP3-like protein (Nix/BNIP3L)-mediated mitophagy, which have been extensively studied (Wu et al., 2017). The downregulation of autophagy and mitophagy has been observed in both eosinophilic and non-eosinophilic nasal polyps (Wang et al., 2022). Research has found that mitochondrial function is significantly impaired in nasal polyps of patients with chronic rhinosinusitis with nasal polyps (CRSwNP). This impairment is characterized by abnormal expression of oxidative phosphorylation complexes, a marked increase in the generation of mitochondrial reactive oxygen species (mtROS), and alterations in mitochondrial morphology, including elongation and disruption of the balance between fusion and fission. These changes in mitochondrial function and morphology may play a crucial role in the pathogenesis of CRSwNP by inducing oxidative stress and cellular damage (Yoon et al., 2020). However, the role of mitophagy in the pathogenesis of CRSwNP is still unclear, and further research is needed to identify precise biomarkers that indicate the occurrence and progression of CRSwNP, in order to develop new clinical strategies for CRSwNP treatment.

This study is based on transcriptome data and aims to identify differentially expressed genes related to mitochondrial autophagy in CRSwNP. Using the Least Absolute Shrinkage and Selection Operator (Lasso) and Boruta algorithms, TFE3 and TP53 were selected as two biomarkers for CRSwNP, providing assistance for the diagnosis and treatment of CRSwNP.

## 2 Materials and methods

### 2.1 Data resource

The GSE136825 and GSE179265 of CRSwNP were scoured from the Gene Expression Omnibus (GEO) database (https://www.ncbi.nlm.nih.gov/geo/). The GSE136825 dataset as training dataset and GSE179265 dataset as validation dataset. The GSE136825 dataset contained 42 polyp samples from CRSwNP and 33 turbinate samples from normal controls, which was based on the GPL20301 platform (Illumina HiSeq 4000). The GSE179265 dataset contained 17 nasal polyps from CRSwNP patients and 7 uncinate processes from normal controls and was based on the GPL24676 platform (Il-lumina HiSeq 6000). In addition, 29 mitophagy-related genes (MRGs) were obtained from the Molecular Signatures Database v7.1 (MSigDB; https://www.gsea-msigdb.org/gsea/msigdb) (REACTOME_MITOPHAGY). 72 MRGs were downloaded from the Kyoto Encyclopedia of Genes and Genomes (KEGG, https://www.kegg.jp/) (hsa04137). After removing duplicates, a total of 81 MRGs were obtained and listed in Supplementary Table S1 IS 03.

### 2.2 Identification of candidate genes

The differentially expressed genes (DEGs) were selected in GSE136825 between CRSwNP and normal controls by the ‘DESeq2 (v.1.36.0)’ R package (Love et al., 2014) The screening thresholds were |log2FC| > 0.5 and P. adjust <0.05. Then, the volcano maps and heat maps of DEGs were represented with the ‘ggplot2 (v 3.3.6)’ (Maag, 2018) and ‘circlize (v. 0.4.15)’ (Gu et al., 2014) R packages. Next, the candidate genes were obtained by overlapping the DEGs and MRGs, and visualized by the ‘ggvenn (v. 0.1.9)’ R package (https://CRAN.R-project.org/package=ggvenn).

### 2.3 Functional enrichment analysis of candidate genes

Based on the Gene Ontology (GO) and Kyoto Encyclopedia of Genes and Genomes (KEGG) databases, the function of candidate genes was enriched by the ‘clusterProfiler (v 4.7.1.001)’ R package (*p* < 0.05) (Yu et al., 2012). Then, the GO and KEGG items were ranked by p from smallest to largest, respectively. Finally, the first 20 items were shown by the ‘treemap (v. 2.4–3)’ R package (https://CRAN.R-project.org/package=treemap).

### 2.4 Protein-protein interaction (PPI) network of candidate genes

To explore the protein interactions of the candidate genes, the PPI network was established based on the STRING database (https://string-db.org) (medium confidence = 0.4) (Szklarczyk et al., 2019), and visualized by Cytoscape (v. 3.8.2) (Smoot et al., 2011).

### 2.5 Machine learning

Here, we used the Lasso regression analysis to screen the signature gene (SGs)-Lasso and the Boruta algorithm to screen the SGs- Boruta for CRSwNP patients, respectively. In Lasso regression analysis, the optimal value of logλ (λ.min) was determined based on the cross-validation 10 times from the ‘glmnet (v 4.1–6)’ R package (Engebretsen and Bohlin, 2019). The Boruta algorithm was performed by the ‘Boruta (v. 8.0.0)’ R package (https://doi.org/10.18637/jss.v036.i11). Then, we overlapped the SGs-Lasso and SGs-Boruta to obtain intersection genes (SGs). Receiver Operating Characteristic (ROC) curves were generated to evaluate the diagnostic performance of SGs. The screening criteria for biomarkers encompass: (1) demonstrating an AUC value greater than 0.70 in both GSE136825 and GSE179265 datasets; (2) exhibiting significant differences between the CRSwNP group and the normal control group in both datasets, with consistent expression trends.

### 2.6 Gene set enrichment analysis (GSEA)

The background gene set “c2. cp.kegg.v7.0. symbols.gmt” was downloaded from the MSigDB database (Subramanian et al., 2005). Spearman’s correlation analysis between biomarkers and all other genes was performed to calculate and rank the correlation coefficient. The GSEA was conducted by the ‘clusterProfiler (v. 4.7.1.001)’ R package (16). The screening criterion was set as |NES|>1 and P. adjust<0.05.

### 2.7 Correlation, friends, and GeneMANIA analysis of biomarkers

The correlations among biomarkers were analyzed based on Spearman’s correlation (*p* < 0.05). Based on the GO analysis result, the functional correlations among biomarkers were analyzed by the ‘GOSemSim (v. 2.22.0)’ R package (Yu, 2020). What’s more, the other genes related to biomarkers and common pathways for them were predicted by the GeneMANIA database (http://genemania.org/).

### 2.8 Backpropagation (BP) neural network

Here, we constructed a BP neural network to evaluate the diagnostic ability of biomarkers by the ‘neuralnet (v. 1.44.2)’ R package (https://CRAN.R-project.org/package=neuralnet). The biomarkers were applied as input vectors. Based on the median expression of biomarkers in all samples of GSE136825, all samples were divided into high- and low-expression groups. The high-expression groups were assigned a value of ‘1’, and the low-expression groups were assigned a value of ‘0’. Then, the BP neural network was constructed. Furthermore, the ROC curve was used to assess the BP neural network.

### 2.9 Infiltrating immune cells analysis

To learn the infiltrating differences of 28 immune cells between CRSwNP and normal controls, the Infiltrating immune cells analysis was conducted by the single sample GSEA (ssGSEA) algorithm of ‘GSVA (v. 1.44.5)’ R package (Hänzelmann et al., 2013). Based on the Wilcox-on test, the differences in immune cells between CRSwNP and normal controls were compared. Additionally, Spearman’s correlations of different immune cells and biomarkers were analyzed (*p* < 0.05).

### 2.10 Potential drug prediction and disease association analysis

The target drugs of biomarkers were predicted based on the Comparative Toxicogenomics Database (CTD) (https://ctdbase.org/). The gene-drug network was constructed based on the Cytoscape (v. 3.8.2) (Smoot et al., 2011). Moreover, to explore the role of biomarkers in other ear-nose-throat (ENT) diseases, we analyzed the correction between ENT diseases and biomarkers on the CTD database.

### 2.11 Clinic samples collection and qPCR

This study involves the use of human tissue samples, and all experiments were conducted in accordance with the principles outlined in the Declaration of Helsinki. The studies involving human participants were reviewed and approved by Qilu Hospital of Shandong University (Qingdao) Medical Ethics Committee (nKYLL-KS-2023161). The patients provided their written informed consent to participate in this study. We collected 5 polyp tissues in CRSwNP patients and 5 turbinate tissues in healthy controls from Qilu Hospital (Qingdao), and these tissues were stored in −80°C freezers. Total RNA was extracted by TRIzol reagent (Ambion, Shanghai, China). cDNA libraries were prepared with 1.5 μg total RNA by SureScript-First-strand-cDNA-synthesCase-kit (Servicebio, Wuhan, China). Then, the qPCR reaction was performed on the CFX Connect Real-Time PCR Detection System (BIO-RAD, U.S.A.), and the reaction system referenced 2xUniversal Blue SYBR Green qPCR Master Mix (Servicebio, Wuhan, China). The primer sequences of biomarkers are listed in Supplementary Table S2. Ultimately, the relative expression levels of biomarkers were normalized by the expression of GAPDH and computed with the 2^−ΔΔCt^ method (Rao et al., 2013).

### 2.12 Statistical analysis

In the study, the statistical analysis of data was employed using the R (v. 4.2.1) package. The Wilcoxon test was applied for difference analysis. All tests were statistically significant with *p* < 0.05.

## 3 Results

### 3.1 A total of eight candidate genes were identified for CRSwNP

**FIGURE 1:**
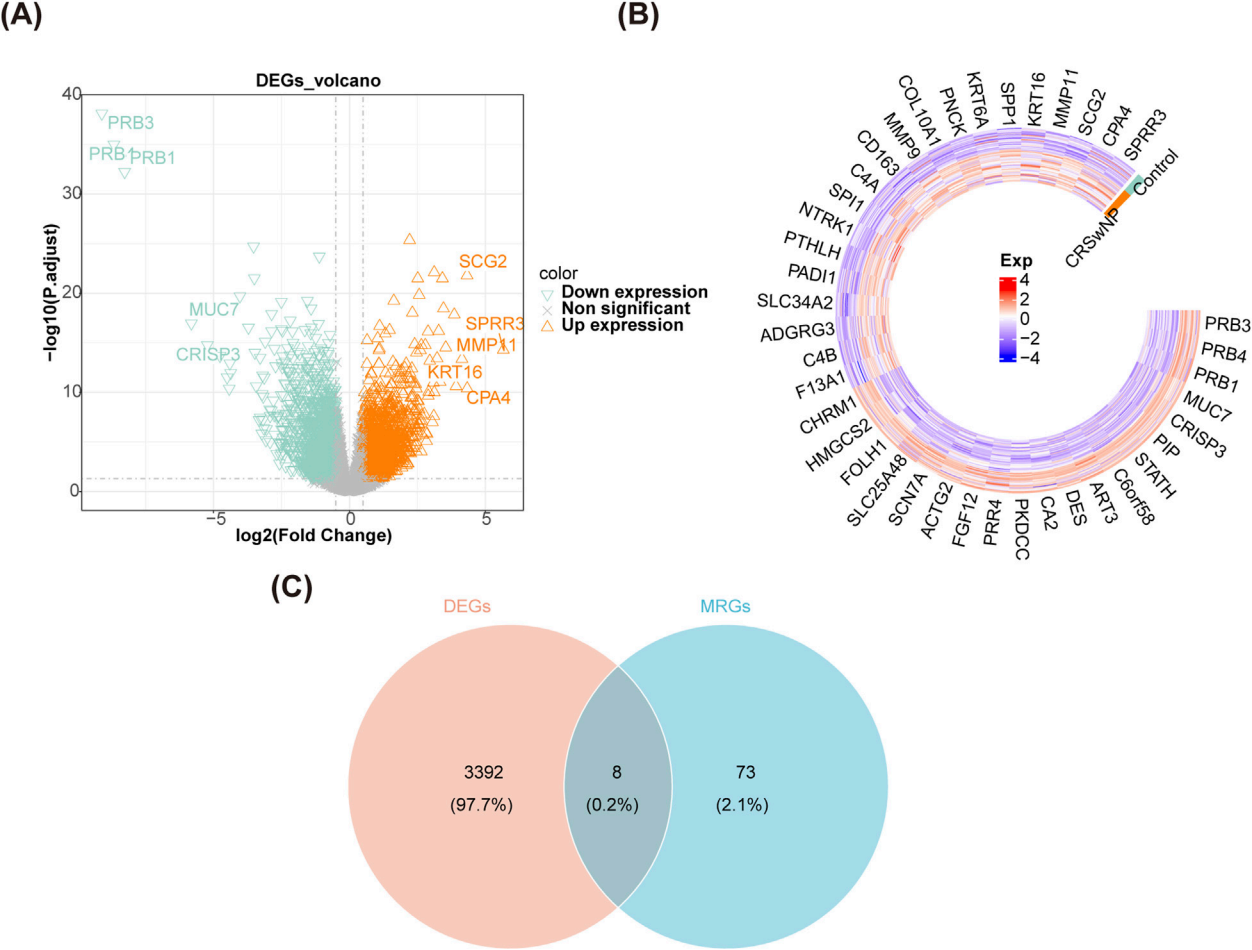
Identification of candidate genes. **(A)** A volcano map of DEGs in GSE136825. **(B)** A heat map of DEGs in GSE136825. **(C)** A Veen diagram of DEGs and MRGs.

**FIGURE 2:**
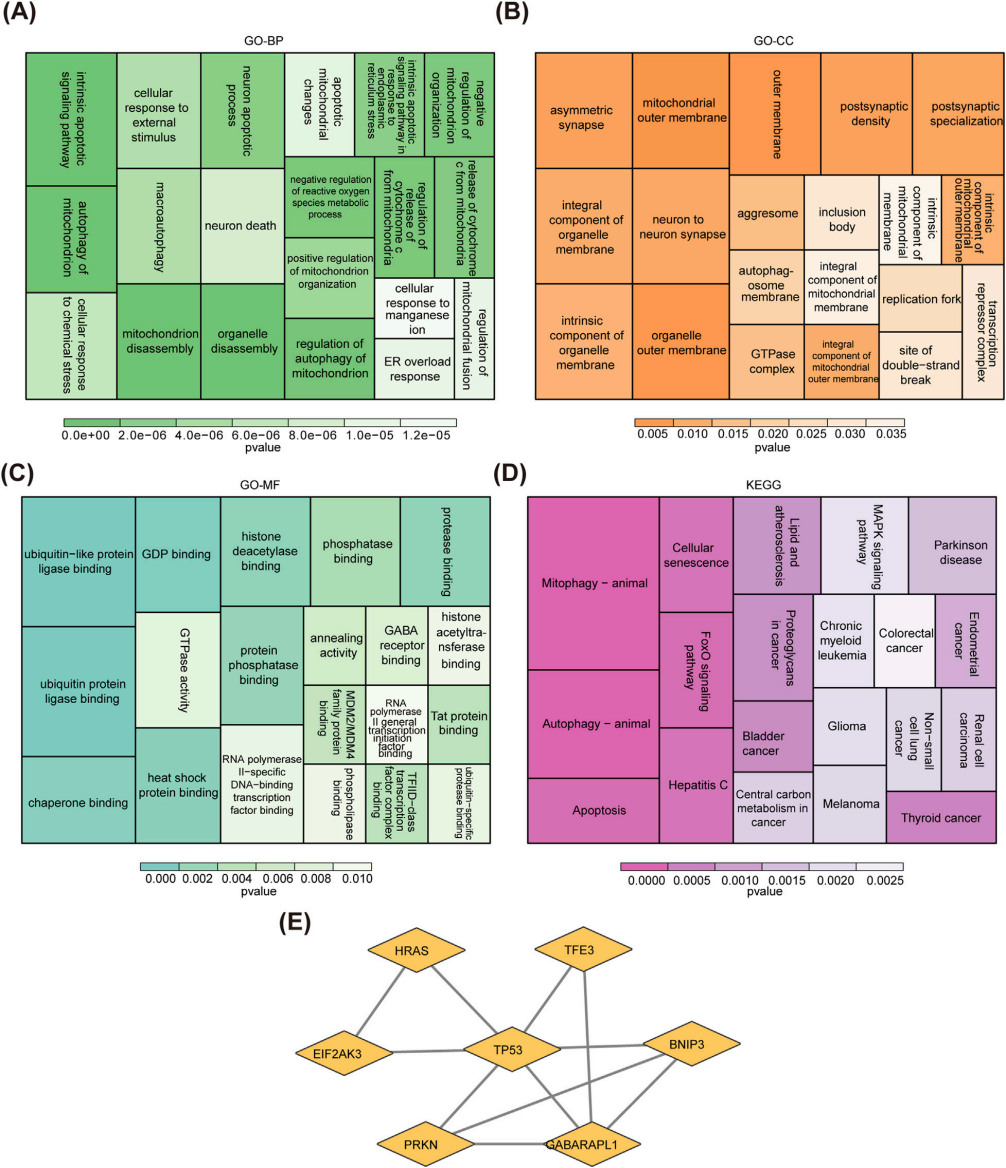
Function enrichment analysis and PPI network of candidate genes. **(A–C)** GO enrichment analysis of candidate genes. **(D)** KEGG enrichment analysis of candidate genes. **(E)** A PPI network of candidate genes. DEGs, differentially expressed genes; MRGs, mitophagy-related genes; GO, gene ontology; KEGG, kyoto encyclopedia of genes and genomes, PPI, protein-protein interaction.

In GSE136825, there were 3,400 DEGs were identified between CRSwNP and normal controls, including 1,904 upregulated genes and 1,496 downregulated genes (Figures 1A, B). After overlapping the DEGs and MRGs, eight intersection genes were obtained as candidate genes for CRSwNP (Figure 1C). Subsequently, the GO and KEGG analyses were applied to explore the function of these candidate genes. There were 631 items in the GO result, including 556 biological processes (BP)-items, 24 cellular components (CC)-items, and 51 molecular functions (MF)-items (Supplementary Table S3). In the BP items, candidate genes were related to autophagy of mitochondrion, mitochondrion disassembly, intrinsic apoptotic signaling pathway, etc. (Figure 2A). In the CC items, candidate genes were associated with the mitochondrial outer membrane, organelle outer membrane, outer membrane, etc. (Figure 2B). In the MF items, candidate genes were linked with the ubiquitin protein ligase binding, ubiquitin-like protein ligase binding, GDP binding, etc. (Figure 2C). The KEGG results showed that a total of 54 KEGG pathways were enriched, such as mitophagy, autophagy, FoxO signaling pathway, etc. (Figure 2D; Supplementary Table S4). Moreover, in order to explore the protein interaction of candidate genes, we constructed a PPI network. There were 7 gene notes (except RRAS) and 11 interaction pairs in this network (Supplementary Table S5). TP53 was located in the center of the network, which interacted with 6 other genes (Figure 2E).

### 3.2 TFE3 and TP53 were determined as biomarkers of CRSwNP

**FIGURE 3:**
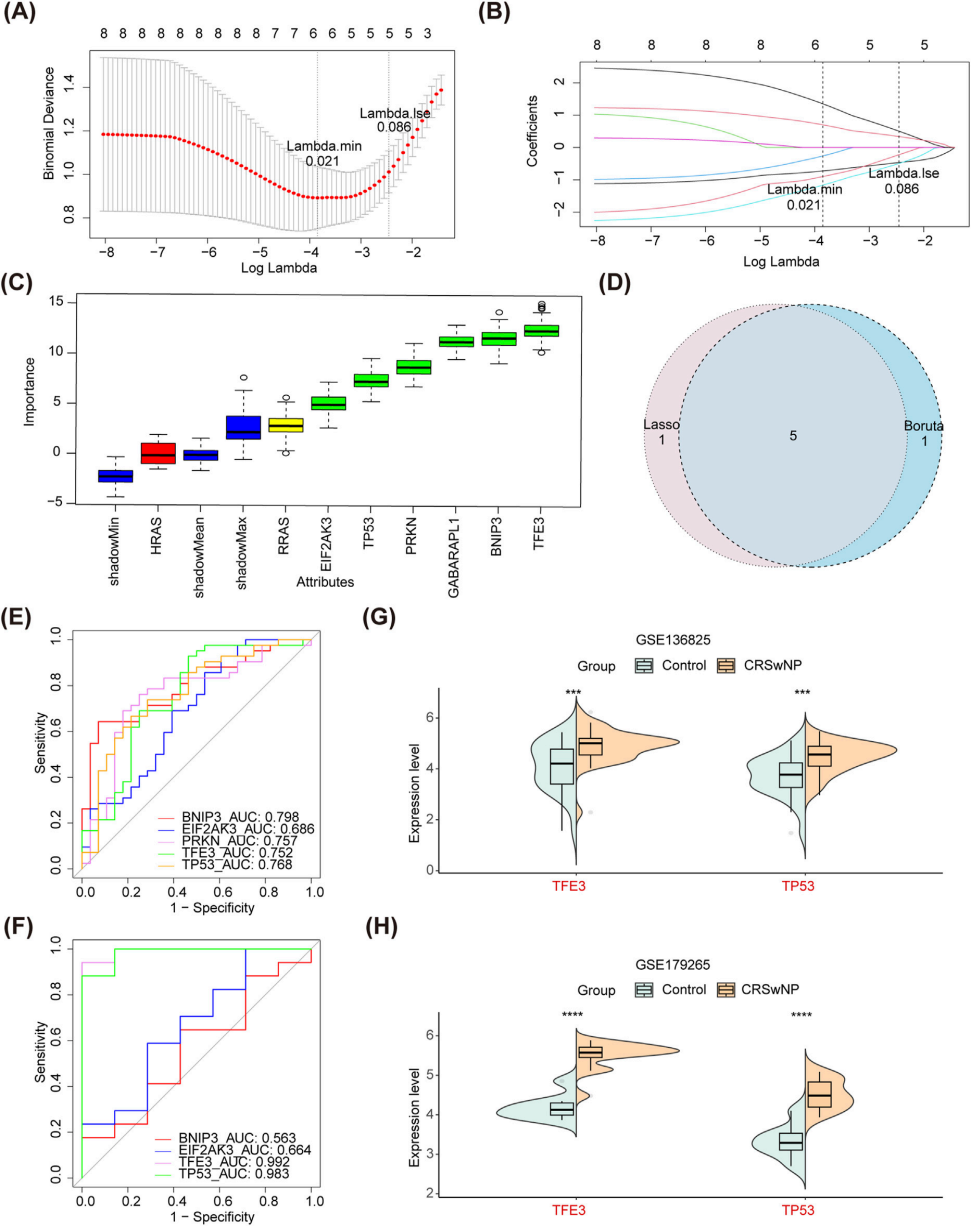
Identification of biomarkers. **(A,B)** Lasso algorithm for SGs-Lasso. **(C)** Boruta algorithm for SGs-Boruta. **(D)** A Veen diagram of SGs-Lasso and SGs-Boruta. **(E)** ROC curves of all SGs in GSE136825. **(F)** ROC curves of all SGs in GSE179265. **(G)** Differential expression analysis of *TFE3* and *TP53* in GSE136825. **(H)** Differential expression analysis of *TFE3* and *TP53* in GSE179265. Lasso, least absolute shrinkage and selection operator; SGs-Lasso, signature genes-Lasso; SGs-Boruta, signature genes-Boruta; ROC, receiver operating characteristic; SGs, signature genes.

In the Lasso regression analysis, the cross-validation was minimal when ‘λ.min = 0.021’, thus 6 SGs-Lasso for CRSwNP were identified (Figures 3A, B). In the Boruta algorithm, 6 SGs-Boruta for CRSwNP were screened (Figure 3C). After overlapping the SGs-Lasso and SGs-Boruta, a sum of 5 intersection genes was determined as SGs, including BNIP3, EIF2AK3, PRKN, TFE3, and TP53 (Figure 3D). In GSE136825, all SGs except EIF2AK3 had AUC values greater than 0.70 (Figure 3E). However, PRKN was not considered because it was not detected in GSE179265. Then, only the AUC of TFE3 and TP53 were above 0.70 (Figure 3F). Moreover, the expression levels of TFE3 and TP53 in CRSwNP were higher than those of normal controls in both GSE136825 and GSE179265 (Figures 3G, H). Therefore, TFE3 and TP53 were identified as biomarkers of CRSwNP.

### 3.3 TFE3 and TP53 had a strong correlation

**FIGURE 4:**
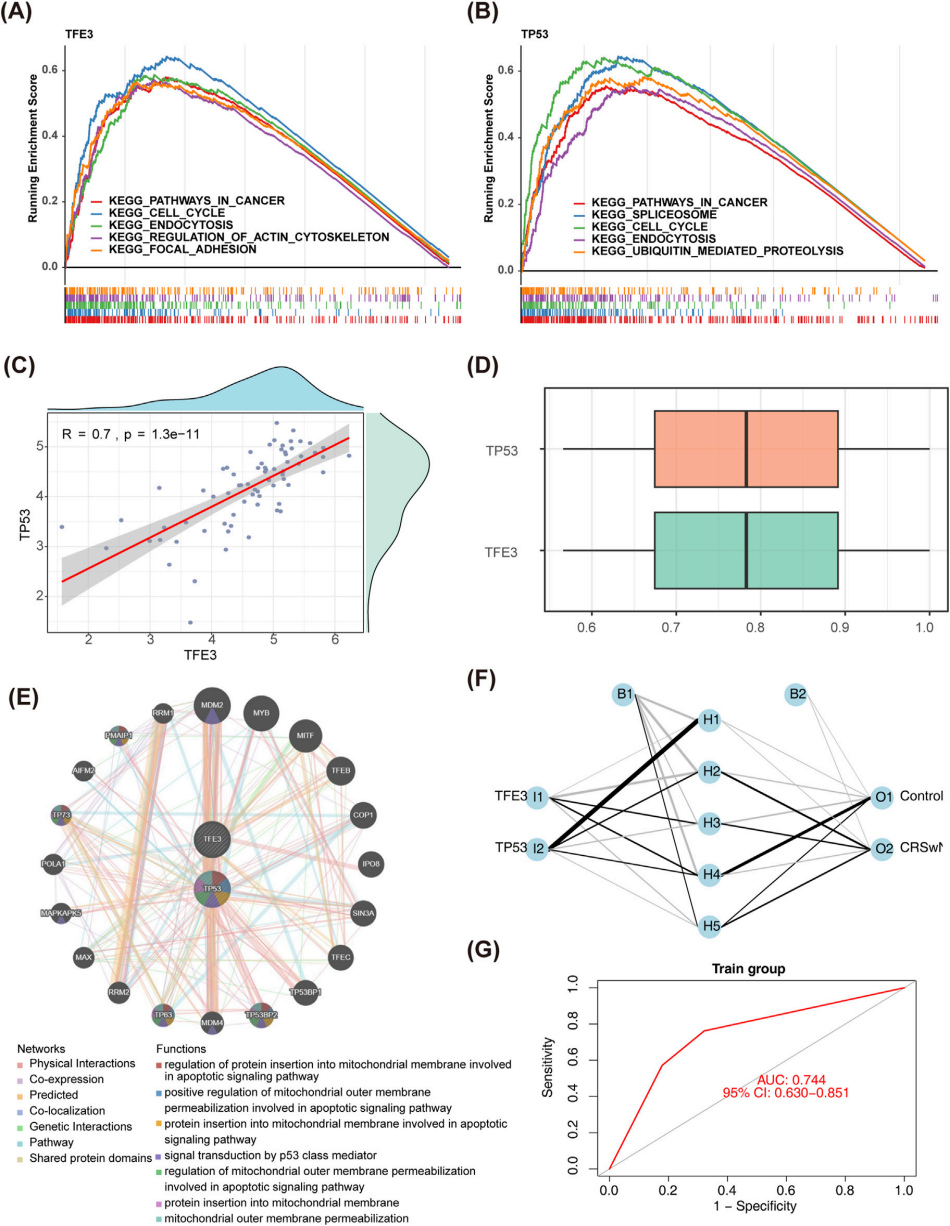
The functional and correlation analysis of biomarkers. **(A,B)** GSEA of *TFE3* and *TP53*. **(C)** Spearman’s correlation analysis between *TFE3* and *TP53*. **(D)** Friends analysis of *TFE3* and *TP53*. **(E)** The GGI network of biomarkers and other related genes. **(F)** A BP neural network of biomarkers in GSE136825. **(G)** ROC curves of BP neural network in GSE136825. GSEA, gene set enrichment analysis; GGI, gene-gene interaction; BP, backpropagation; ROC, receiver operating characteristic.

Here, we explored further the KEGG pathway involved in biomarkers. Interestingly, TFE3 and TP53 were co-enriched in the ‘cancer pathway’, ‘cell cycle’, ‘endocytosis’, etc. (Figures 4A, B). The correlation coefficient was 0.70 between TFE3 and TP53 (*p* < 0.001) (Figure 4C). The functional similarity of TFE3 and TP53 was also higher, which was close to 0.8 (Figure 4D). Furthermore, 20 genes with strong interaction relationships with biomarkers were obtained in the GeneMANIA database. MDM2, MDM4, TP53BP2, TP53BP1, etc. with biomarkers that had high correlations, and they were enriched in mitochondrial and apoptosis-related signaling pathways (Figure 4E). In addition, the BP neural network was generated (Figure 4F). The AUC value of this network was 0.74, which indicated that the biomarkers had excellent abilities to distinguish CRSwNP and normal controls (Figure 4G).

### 3.4 CD56^bright^ natural killer cell had the highest correlation with biomarkers

**FIGURE 5:**
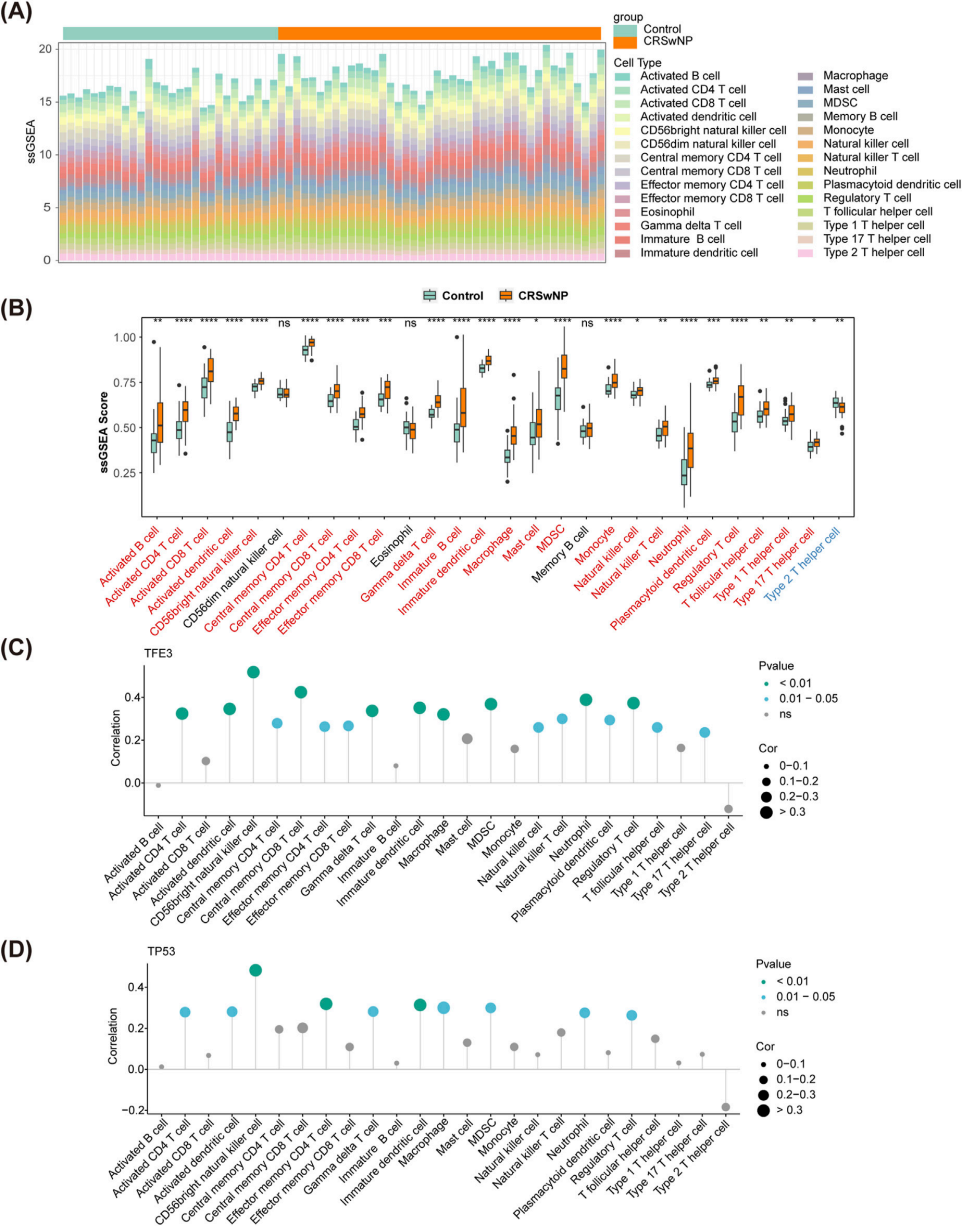
Immune cell infiltration in GSE136825. **(A)** The fraction of 28 immune cells in CRSwNP and normal controls. **(B)** The differences of immune infiltration between CRSwNP and normal controls. **(C,D)** Spearman’s correlation analyses between the biomarkers and different immune cells. CRSwNP, chronic rhinosinusitis with nasal polyps; ‘*’, ‘**’, ‘***’, and ‘****’ represent *p* < 0.05, *p* < 0.01, *p* < 0.001, *p* < 0.0001; ‘ns’ represents no significant difference.

Based on the ssGSEA algorithm, the scores of 28 immune cells in CRSwNP and normal controls were calculated, and the proportion of immune cells was shown in Figure 5A. Then, the ssGSEA scores were compared between CRSwNP and normal controls. Thereinto, 25 immune cells had significant differences between the two groups (except CD56dim natural killer cell, Eosinophil, and Memory B cell) (Figure 5B). Among them, only type 2 T helper cells in CRSwNP had lower scores than those in normal controls. Importantly, CD56bright natural killer cells had the highest correlation with TFE3 (cor = 0.52, *p* < 0.01) and TP53 (cor = 0.48, *p* < 0.01) (Figures 5C, D). Moreover, macro-phage and immature dendritic cells had significant correlations with biomarkers (|cor|>0.3, *p* < 0.05) (Supplementary Tables S6, S7).

### 3.5 A sum of 43 drugs and 71 ENT diseases related to biomarkers were predicted

**FIGURE 6:**
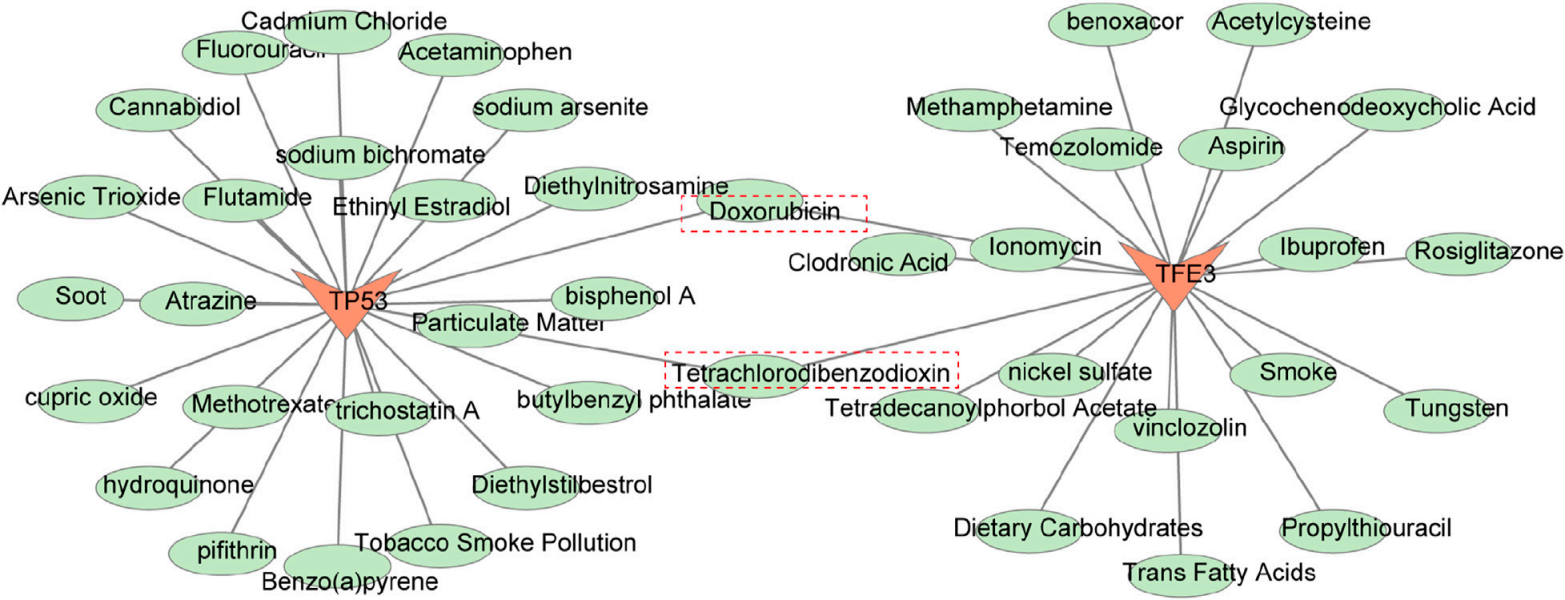
Drug-gene network.

As mentioned earlier, the expression levels of both TFE3 and TP53 were upregulated in CRSwNP, thus the drugs that could make the biomarkers downregulated were selected as potential therapeutic agents for CRSwNP. We screened 20 drugs with TFE3 and 25 drugs with TP53 from the CTD database, and a total of 43 drugs were obtained (Supplementary Table S8). Among them, Doxorubicin and Tetrachlorodibenzodioxin were associated with both TFE3 and TP53 (Figure 6). Moreover, 71 ENT diseases associated with biomarkers were predicted (Supplementary Tables S9, S10), such as hearing loss, sensorineural, tinnitus, etc. (Supplementary Figure S1).

### 3.6 Verification of TFE3 and TP53 in clinical samples by qPCR

**FIGURE 7:**
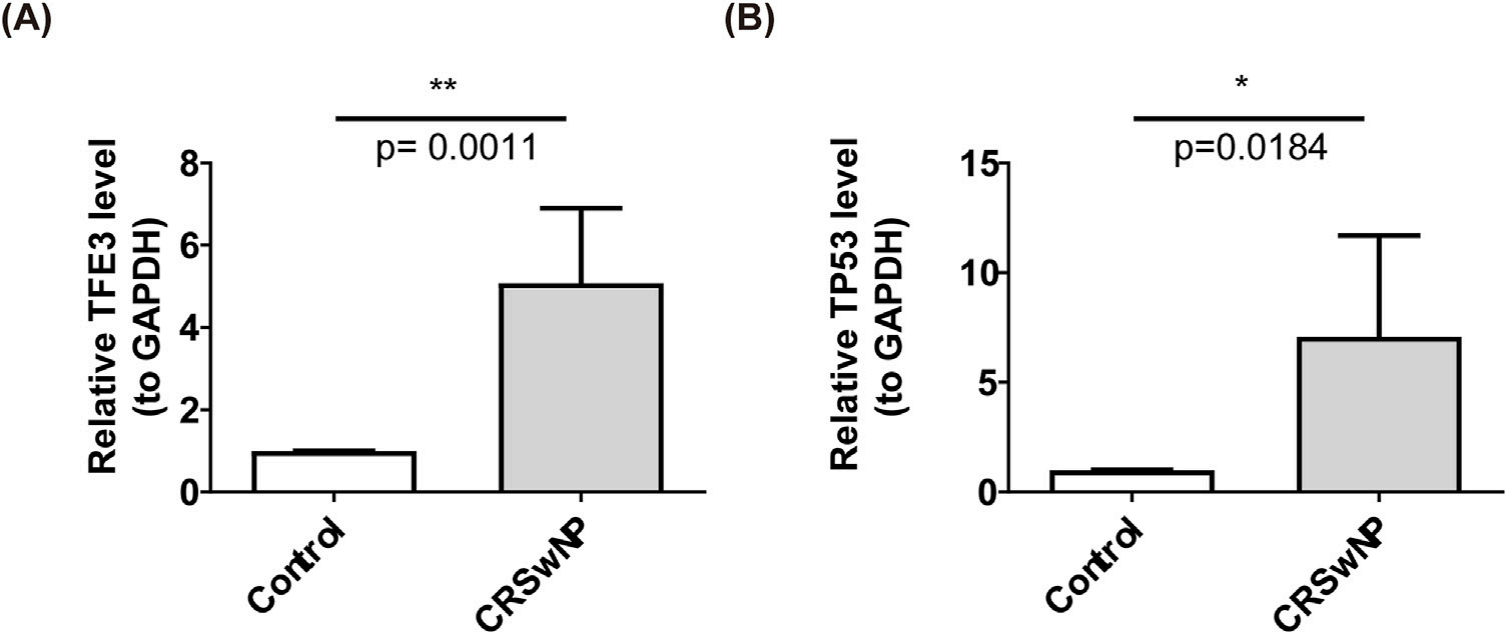
The qPCR results of biomarkers, **(A)** TFE3 **(B)** TP53. ‘*’ represent *p* < 0.05 and ‘**’ represent *p* < 0.01.

Next, we performed qPCR analyses after collecting clinic samples. As shown in Figures 7A, B, compared with healthy controls, the expression levels of TFE3 and TP53 in polyp tissue of CRSwNP were increased (*p* < 0.05). qPCR results were consistent with Figures 3G, H.

## 4 Discussion

In patients with CRSwNP, the nasal and sinus mucosal epithelial barrier is impaired and exhibits abnormal remodeling (Yan et al., 2013; Radajewski et al., 2019), resulting in the infiltration of numerous pathogens, toxins, and allergens into the submucosal tissue, triggering an inflammatory response, ultimately leading to the remodeling of nasal mucosal tissue and the formation of nasal polyps (Lan et al., 2018). The diagnosis of CRSwNP faces challenges primarily due to the lack of specific biomarkers and an incomplete understanding of the underlying pathological mechanisms. This situation results in a reliance on symptoms and imaging examinations for diagnosis, which often fail to accurately reflect the biological essence of the disease. Furthermore, the pathological mechanisms of CRSwNP are complex and diverse, involving various immune cells and molecular pathways, but the specific roles of these mechanisms remain unclear, limiting the development of effective treatment methods. Understanding the molecular mechanisms by which nasal mucosal epithelial cells participate in repair, proliferation, and mucociliary differentiation under normal and pathological conditions can provide new insights into improving the epithelial barrier function in chronic rhinosinusitis with nasal polyps.

In this study, we identified two novel biomarkers (TFE3 and TP53) related to mitochondrial energy metabolism through lasso and Boruta algorithms. The expression levels of these biomarkers showed significant differences between patients with CRSwNP and controls, indicating their clinical significance. Infiltrating immune cells analysis showed that CD56bright natural killer cells had highly positive correlations with biomarkers. Also, the two biomarkers were associated with hearing loss, sensorineural, tinnitus, etc. The significance of these two biomarkers lies in their association with the diagnosis and treatment of CRSwNP, highlighting their potential as important indicators in clinical practice.

In normal physiological conditions, TFE3 is regulated by phosphorylation by mTORC1, binding to 14-3-3 proteins and remaining in the cytoplasm (Martina et al., 2014; Mar et al., 2012). However, during starvation, mTORC1 activity decreases, TFE3 is dephosphorylated and translocates into the nucleus, activating the transcription of genes that regulate lysosome biogenesis, function, and autophagy, maintaining cellular nutrient and energy balance. Meanwhile, TFE3 collaborates with other members of the MiT-TFE family, such as MITF, to regulate the biogenesis of lysosomes and the expression of genes involved in starvation-induced autophagic responses (Ramire et al., 2021; Möller et al., 2019; Vu et al., 2021). Research has indicated that nuclear expression of TFE3 has also been observed in certain types of pancreatic tumors, suggesting that TFE3 may be implicated in the pathogenesis and progression of these tumors. However, it is important to note that the specific mechanisms by which TFE3 operates in tumors require further investigation. Overall, TFE3, as a transcription factor, plays a crucial role in regulating cellular growth, differentiation, and metabolism, and its aberrant expression may be closely associated with the onset and progression of various cancers (Vu et al., 2021). Previous studies have demonstrated that TFE3 is a transcription factor expressed in melanoma, which is closely associated with MITF, and plays a crucial role in regulating the sensitivity of cells to iron-induced cell death (Dias et al., 2024). Research indicates that patients with TFE3-positive renal cell carcinoma (RCC) are more prone to lymph node and distant metastases, exhibit higher tumor nuclear grades and clinical stages, and demonstrate shorter progression-free survival. This suggests that TFE3 positivity serves as an independent prognostic factor for poor outcomes in RCC (Dong et al., 2022). Given the critical role of TEF3 in tumorigenesis and progression, it may serve as a potential therapeutic target for cancer treatment. By inhibiting the activity or expression of TEF3, it may be possible to suppress tumor cell proliferation, migration, and angiogenesis, thereby decelerating tumor progression and enhancing patient survival rates. TFE3 is a transcription factor associated with cell growth, differentiation, and apoptosis, and it may play an important role in the pathogenesis of sinus inflammation.

The TP53 gene encodes the p53 protein, which is one of the major regulatory factors for cell division and cell death. It can respond to various stresses, including radiation, toxic substances, hypoxia, excessive production of reactive oxygen species (ROS), and uncontrolled cell cycle, and become activated and stabilized (Capuozzo et al., 2022) The p53 protein is capable of effectively inducing cell cycle arrest, DNA repair, cellular senescence, apoptosis, and autophagy. Research has found that TP53 primarily fine-tunes the cell cycle progression and apoptosis mechanisms by regulating the expression of multiple microRNAs. TP53 is able to inhibit genes that promote cell cycle progression (such as CDK4, CDK6, CCND1, CCNE2, and E2F3), while also suppressing the expression of anti-apoptotic genes (such as BCL-2 and survivin), thereby preventing abnormal cell proliferation and promoting cell apoptosis (McCubrey et al., 2017). Previous studies have observed the significant role of TP53 in chronic sinusitis, particularly in chronic rhinosinusitis with nasal polyps (CRSwNP). Mutations in TP53 within the context of CRSwNP may lead to a loss or attenuation of its tumor suppressor functions, thereby increasing the risk of polyp formation or promoting the growth of nasal polyps (Wan et al., 2023; Brar et al., 2023; Huang and Liu, 2023). These findings provide important context for our research and motivate us to further explore the potential value of TP53 as a biomarker for CRSwNP.

Despite the detection of TP53 gene mutations in approximately 50% of tumor samples, the presence of these mutations reveals more about the complexity of tumorigenesis than a singular causal relationship (Kennedy and Lowe, 2022; Levine, 2020; Levine, 2021). The positive selection of these mutations can be attributed to their ability, in certain contexts, to confer survival and proliferative advantages to tumor cells. However, it is noteworthy that not all TP53 mutations result in a complete loss of protein function; some mutations may even endow the protein with novel functions (termed gain-of-function), thereby uniquely facilitating tumor progression. Furthermore, the impact of TP53 mutations on tumor behavior is influenced by various factors, including the specific type of mutation, cellular context, and tumor microenvironment.

TP53, as a crucial tumor suppressor gene, plays a central role in maintaining cellular genomic stability and preventing tumor formation. However, mutations in TP53 are closely associated with the development of various tumors and may even be involved in the pathological processes of diseases such as chronic rhinosinusitis with nasal polyps (CRSwNP). In the context of numerous malignancies, mutations in TP53 occur frequently, leading to a loss of function that subsequently promotes the aberrant proliferation and dissemination of tumor cells. Furthermore, TP53 profoundly influences the immune cell landscape within the tumor microenvironment (TME), particularly exerting a significant impact on the reprogramming and polarization processes of macrophages (El-Arabey et al., 2020). These findings not only elucidate the complex regulatory mechanisms of TP53 within the tumor immune microenvironment but also suggest that TP53 and its associated regulatory pathways may serve as potential therapeutic strategies for specific cancer types. By precisely modulating the status of TP53, we anticipate the possibility of influencing the infiltration patterns of immune cells within the tumor microenvironment, thereby opening new avenues for improving clinical outcomes for patients.

In the existing research context, investigations directly linking TFE3 and CRSwNP are particularly scarce. This study explores the potential relationship between TFE3 and CRSwNP, and preliminarily reveals that TFE3 may serve as a genetic factor playing a critical role in the occurrence and development of CRSwNP. This finding provides new perspectives and clues for further understanding the pathogenesis of CRSwNP and developing related therapeutic strategies. Regarding TP53, although its role in CRS has been reported, this study further elucidates the specific mechanisms of TP53 in CRSwNP and its potential as a biomarker. The functional mechanisms of these two genetic factors require further investigation to comprehensively understand their roles in the pathological processes of CRSwNP.

In the context of existing research, investigations directly linking TFE3 and CRSwNP are particularly scarce. This study explores the potential connection between TFE3 and CRSwNP, and preliminarily reveals that TFE3 may serve as a genetic factor playing a critical role in the occurrence and development of CRSwNP. This finding provides new perspectives and clues for further understanding the pathogenesis of CRSwNP and for developing related therapeutic strategies. Regarding TP53, although its role in CRS has been documented, this study delves deeper into the specific mechanisms of TP53 in CRSwNP and its potential as a biomarker. The functional mechanisms of these two genetic factors require further in-depth investigation to comprehensively understand their roles in the pathological processes of CRSwNP.

TFE3 is a transcription factor associated with cell growth, differentiation, and apoptosis, and it may play an important role in the pathogenesis of sinus inflammation. TP53 is a tumor suppressor gene, and its mutations are associated with the development of various tumors, potentially playing a role in the pathogenesis of CRSwNP. Additionally, by predicting the targeted drugs of biomarkers and analyzing their associations with other otorhinolaryngological diseases, we speculate that TFE3 and TP53 genes may serve as potential drug targets and be involved in the occurrence and development of various otorhinolaryngological diseases. Future studies will further explore the functional mechanisms of these two genetic factors through methods such as gene knockout and overexpression, aiming to provide new strategies for the diagnosis, treatment, and prevention of CRSwNP.

Particularly noteworthy is the observed correlation between CD56bright natural killer cells and the transcription factors TFE3 and TP53. This association suggests a potential interaction between specific immune cell subtypes and key regulatory genes, providing new insights into the molecular mechanisms underlying chronic rhinosinusitis with nasal polyps (CRSwNP). Furthermore, our analysis revealed significant correlations between macrophages, immature dendritic cells, and specific biomarkers. This implies that these immune cell populations may play a role in modulating molecular pathways associated with CRSwNP, thereby opening new avenues for targeted therapeutic interventions (Poli et al., 2009; Jacobs et al., 2001). Given their significance, we aim to investigate the potential association between biomarkers and CD56. The expression of CD56 is related to immune activation and immune surveillance, serving as a crucial tool for monitoring immune responses and disease progression. By exploring the relationship between CD56 and TFE3/TP53, we can delve into the complex interactions between immune status and tumor biology. Of particular interest was the observed correlation between CD56bright natural killer cells and the transcription factors TFE3 and TP53. This association suggests a potential interplay between specific immune cell subtypes and key regulatory genes, shedding light on novel avenues for understanding CRSwNP at the molecular level. Moreover, our analysis identified significant correlations between macrophages, immature dendritic cells, and specific biomarkers. This implies a potential involvement of these immune cell populations in the modulation of CRSwNP-related molecular pathways, opening avenues for targeted therapeutic interventions.

In summary, this study relied on the GEO database and applied a series of bioinformatics methods to identify mitochondrial autophagy-related biomarkers TFE3 and TP53 in CRSwNP. They were enriched in cancer pathways, cell cycle, and endocytosis. Additionally, a significant correlation was found between macrophages and immature dendritic cells with the biomarkers. Drugs related to the biomarkers and ear, nose, and throat diseases such as hearing loss and sensorineural tinnitus were predicted. The findings provide a new theoretical basis for the diagnosis, treatment, and pathogenesis of CRSwNP. However, limitations exist due to the reliance on database analysis and the lack of experimental validation of specific molecular mechanisms. Further exploration is necessary to understand the specific mechanisms between mitochondrial autophagy and CRSwNP, in order to provide more valuable clinical treatment references and foundations.

## Data Availability

The data presented in the study are deposited in the Gene Expression Omnibus (GEO) database (https://www.ncbi.nlm.nih.gov/), accession number GSE136825 and GSE179265. The Molecular Signatures Database v7.1 (MSigDB; https://www.gsea-msigdb.org/gsea/msigdb) (REACTOME_MITOPHAGY). The72 MRGs were deposited in the Kyoto Encyclopedia of Genes and Genomes (KEGG, https://www.kegg.jp/) (hsa04137).
