# Supplementary material for: TFE3 and TP53 were novel diagnostic biomarkers related to mitochondrial autophagy in chronic rhinosinusitis with nasal polyps
Source: Front Genet. 2024 Oct 8;15:1423778. doi: 10.3389/fgene.2024.1423778 (PMC11493635; doi:10.3389/fgene.2024.1423778)
Supplement: Supplementary file 1 [file DataSheet3.ZIP › 原始数据-上传frontiers in genetics/02_result/02_Venn/fig2-2B.GO_CC_treemap_plot.pdf]

GO-CC

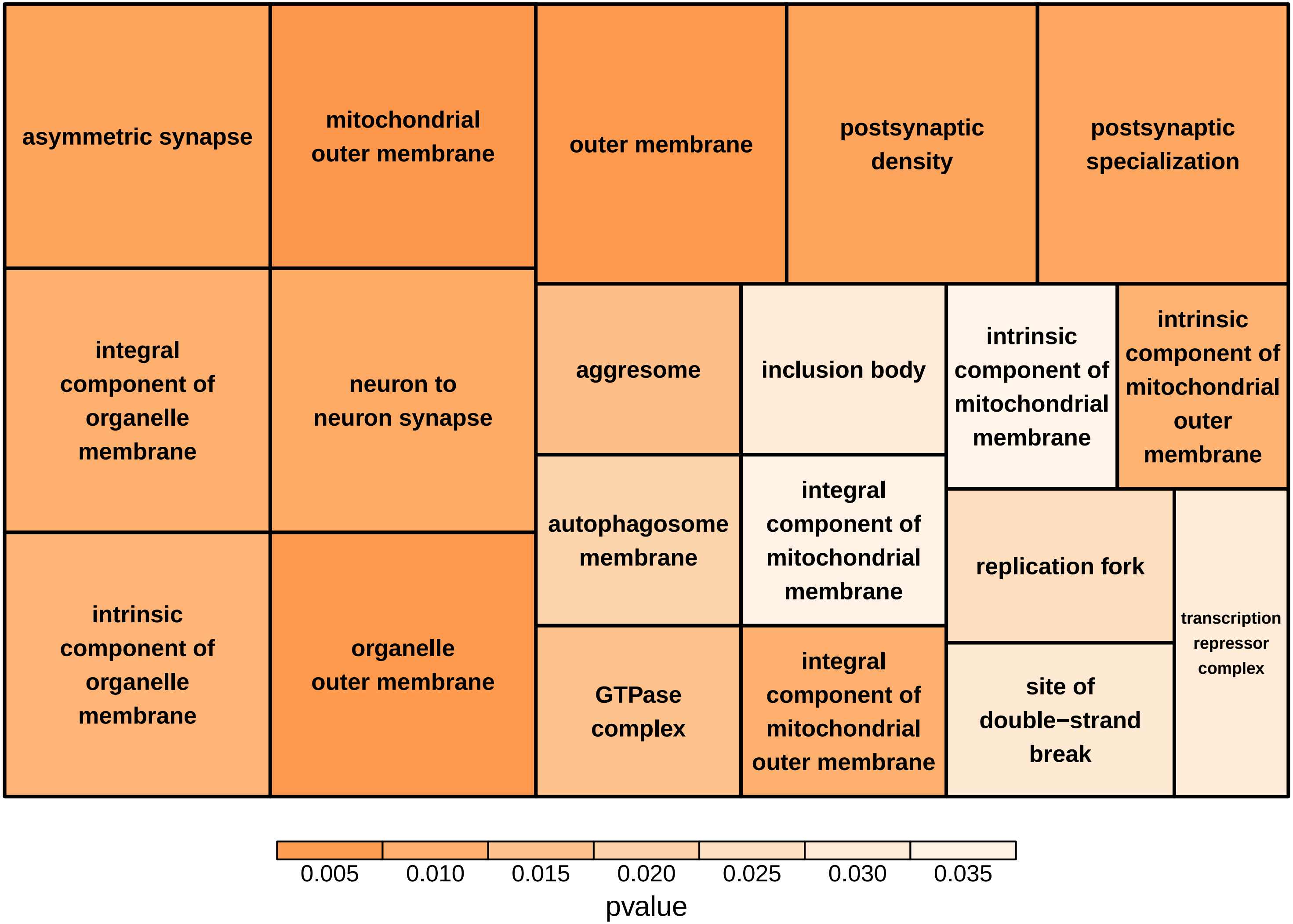
asymmetric synapse
mitochondrial outer membrane
outer membrane
postsynaptic density
postsynaptic specialization
integral component of organelle membrane
neuron to neuron synapse
aggresome
inclusion body
intrinsic component of mitochondrial membrane
intrinsic component of mitochondrial outer membrane
autophagosome membrane
integral component of mitochondrial membrane
replication fork
transcription repressor complex
intrinsic component of organelle membrane
organelle outer membrane
GTPase complex
integral component of mitochondrial outer membrane
site of double−strand break
0.005
0.010
0.015
0.020
0.025
0.030
0.035
pvalue
