# Supplementary material for: TFE3 and TP53 were novel diagnostic biomarkers related to mitochondrial autophagy in chronic rhinosinusitis with nasal polyps
Source: Front Genet. 2024 Oct 8;15:1423778. doi: 10.3389/fgene.2024.1423778 (PMC11493635; doi:10.3389/fgene.2024.1423778)
Supplement: Supplementary file 1 [file DataSheet3.ZIP › 原始数据-上传frontiers in genetics/02_result/06_GSEA/02.GSEA/01.TFE3.pdf]

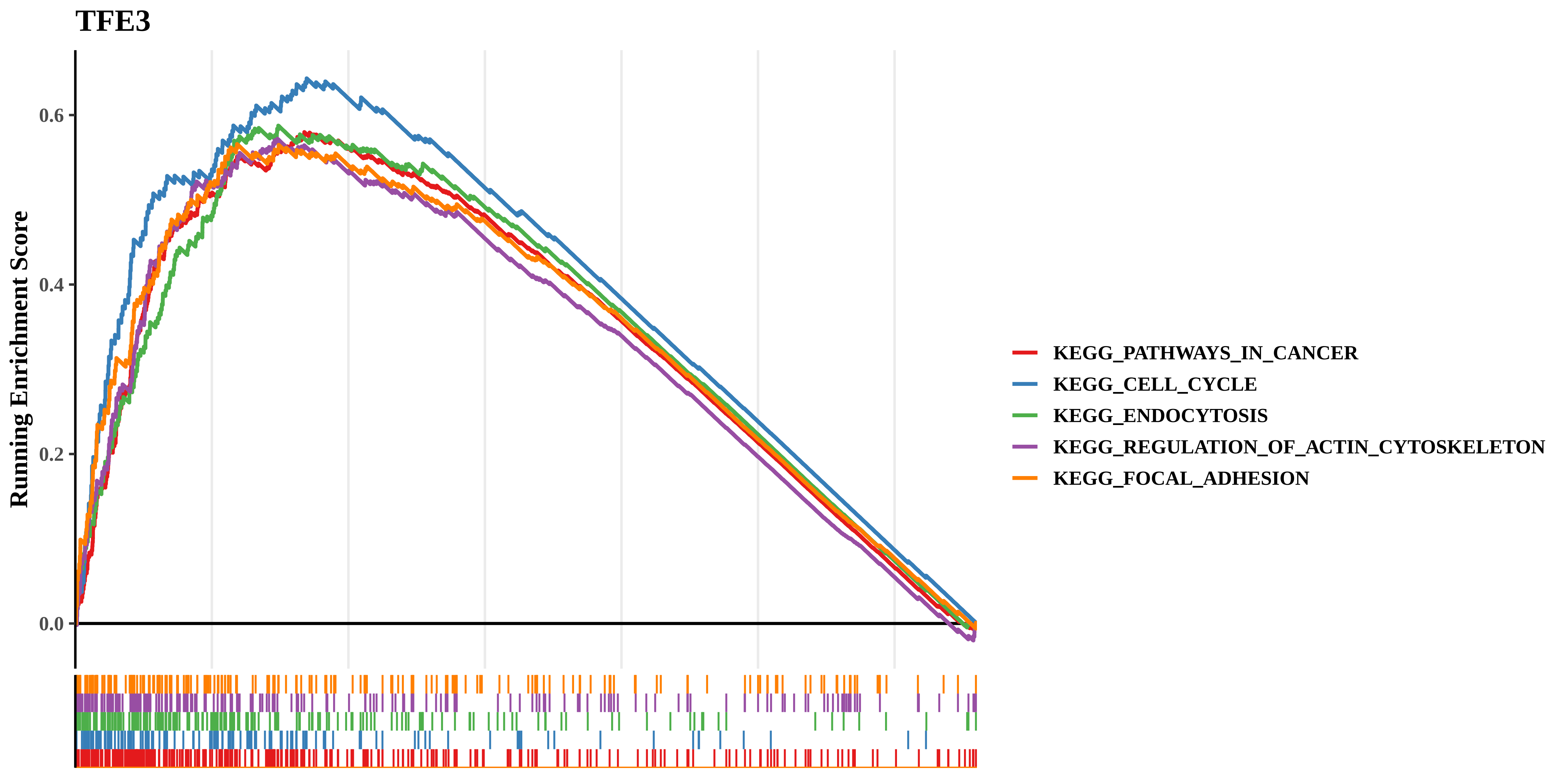
TFE3
Running Enrichment Score
0.6
0.4
0.2
0.0
KEGG_PATHWAYS_IN_CANCER
KEGG_CELL_CYCLE
KEGG_ENDOCYTOSIS
KEGG_REGULATION_OF_ACTIN_CYTOSKELETON
KEGG_FOCAL_ADHESION
