# Supplementary material for: TFE3 and TP53 were novel diagnostic biomarkers related to mitochondrial autophagy in chronic rhinosinusitis with nasal polyps
Source: Front Genet. 2024 Oct 8;15:1423778. doi: 10.3389/fgene.2024.1423778 (PMC11493635; doi:10.3389/fgene.2024.1423778)
Supplement: Supplementary file 1 [file DataSheet3.ZIP › 原始数据-上传frontiers in genetics/02_result/08_GeneMAINA/fig8.GeneMANIA.pdf]

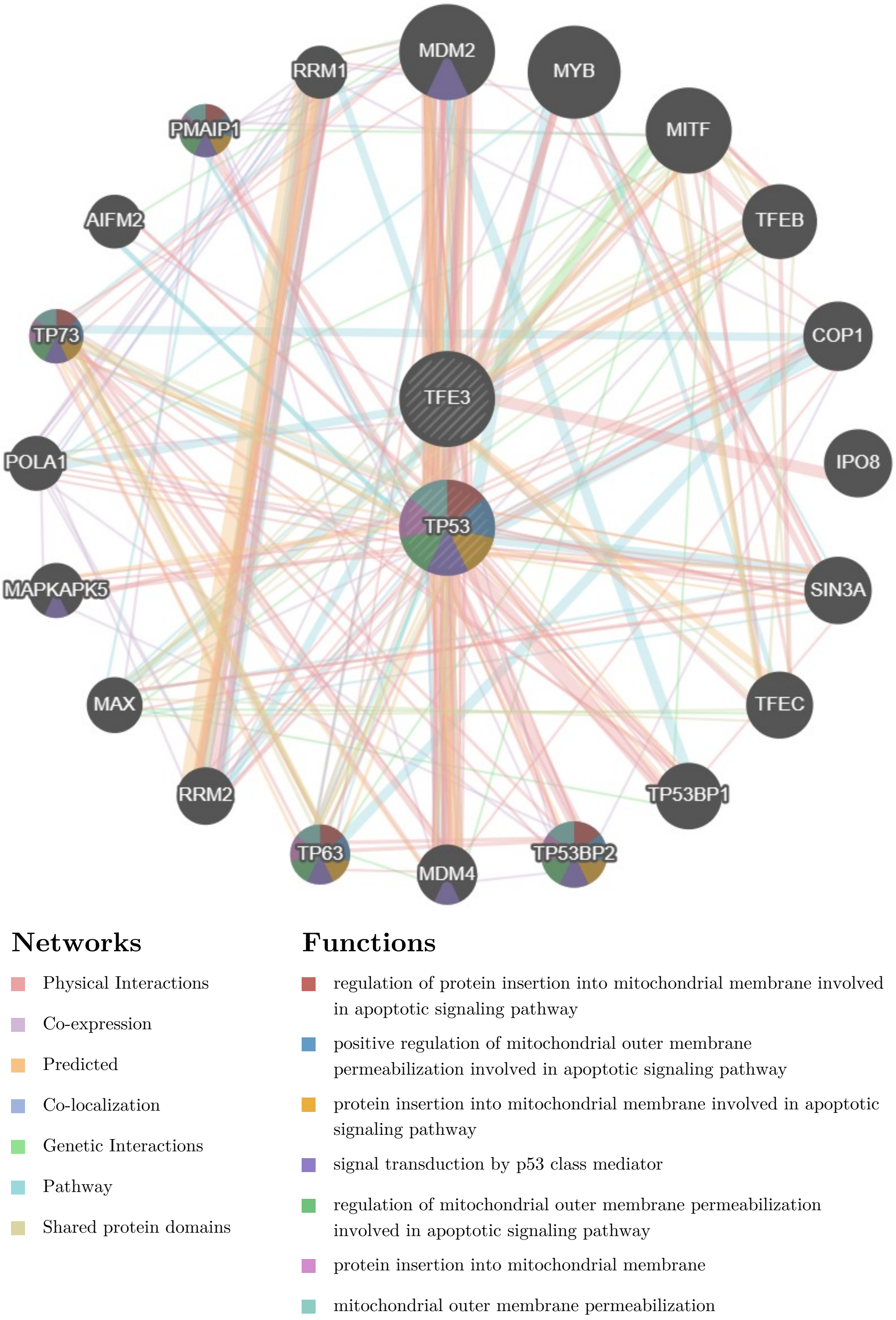
MDM2
MYB
RRM1
PMAIP1
MITF
AIFM2
TFEB
TP73
COP1
TFE3
POLA1
IPO8
TP53
MAPKAPK5
SIN3A
MAX
TFEC
RRM2
TP53BP1
TP63
TP53BP2
MDM4
Networks
Physical Interactions
Co-expression
Predicted
Co-localization
Genetic Interactions
Pathway
Shared protein domains
Functions
regulation of protein insertion into mitochondrial membrane involved in apoptotic signaling pathway
positive regulation of mitochondrial outer membrane permeabilization involved in apoptotic signaling pathway
protein insertion into mitochondrial membrane involved in apoptotic signaling pathway
signal transduction by p53 class mediator
regulation of mitochondrial outer membrane permeabilization involved in apoptotic signaling pathway
protein insertion into mitochondrial membrane
mitochondrial outer membrane permeabilization
