# Supplementary material for: TFE3 and TP53 were novel diagnostic biomarkers related to mitochondrial autophagy in chronic rhinosinusitis with nasal polyps
Source: Front Genet. 2024 Oct 8;15:1423778. doi: 10.3389/fgene.2024.1423778 (PMC11493635; doi:10.3389/fgene.2024.1423778)
Supplement: Supplementary file 1 [file DataSheet3.ZIP › 原始数据-上传frontiers in genetics/02_result/09_Disease/fig9-2.TP53_CTD.pdf]

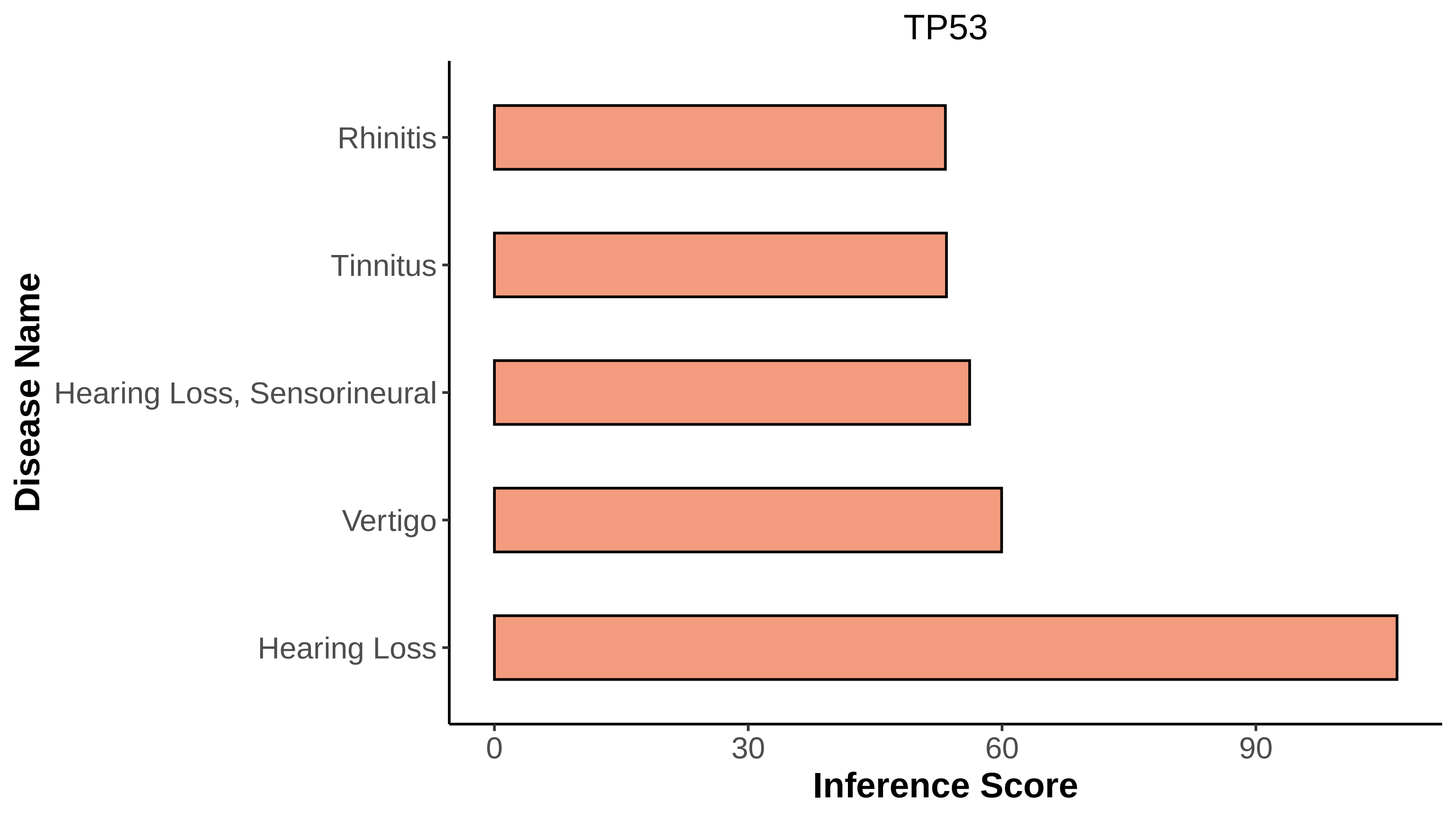
TP53
Disease Name
Rhinitis
Tinnitus
Hearing Loss, Sensorineural
Vertigo
Hearing Loss
0
30
60
90
Inference Score
