# Supplementary figures and images for: TFE3 and TP53 were novel diagnostic biomarkers related to mitochondrial autophagy in chronic rhinosinusitis with nasal polyps
Source: Front Genet. 2024 Oct 8;15:1423778. doi: 10.3389/fgene.2024.1423778 (PMC11493635; doi:10.3389/fgene.2024.1423778)

DEGs\_volcano

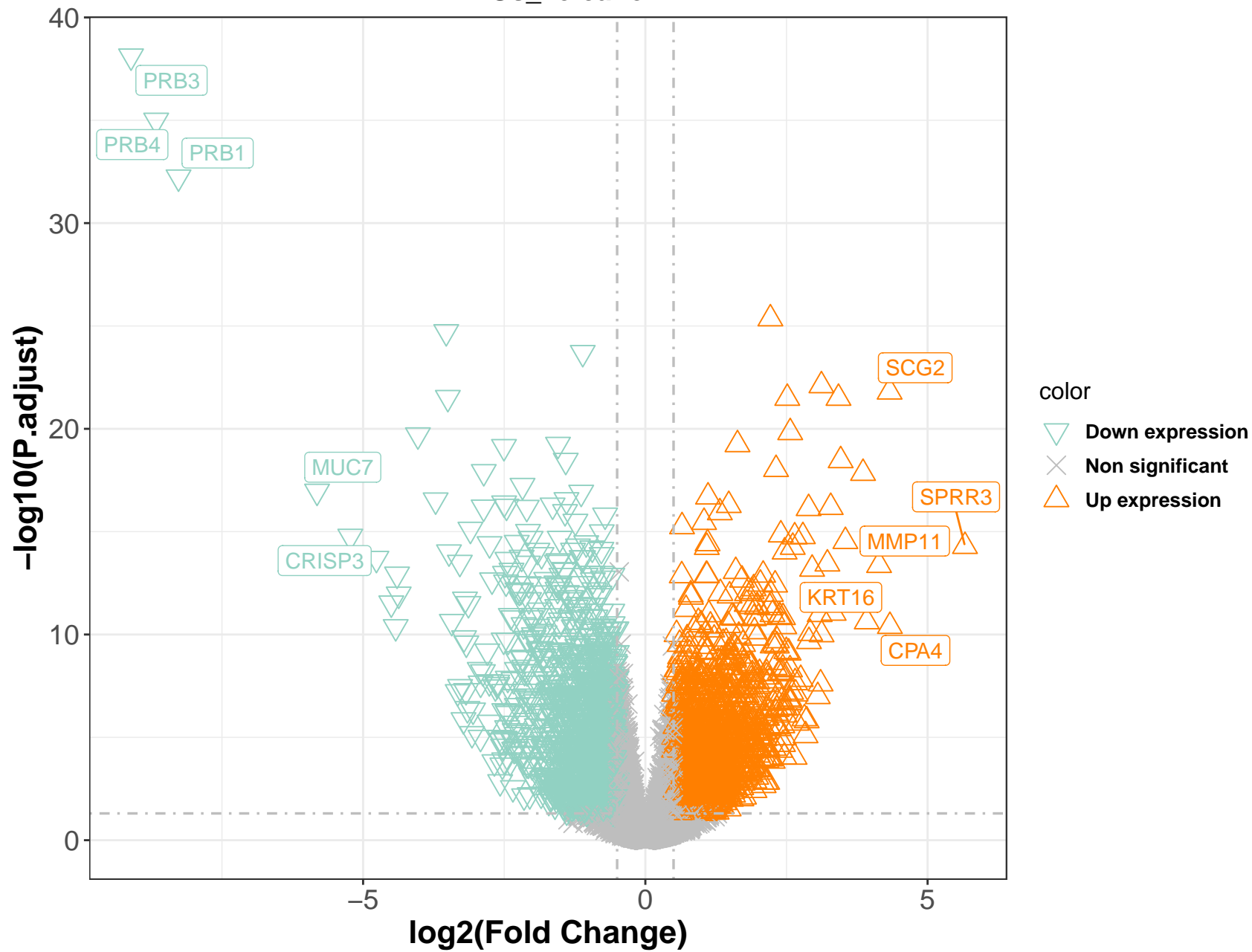

Supplement: Supplementary file 1 [file DataSheet3.ZIP › 原始数据-上传frontiers in genetics/02_result/01_DEG/fig1-1.DEG_volcano_symbol.pdf]

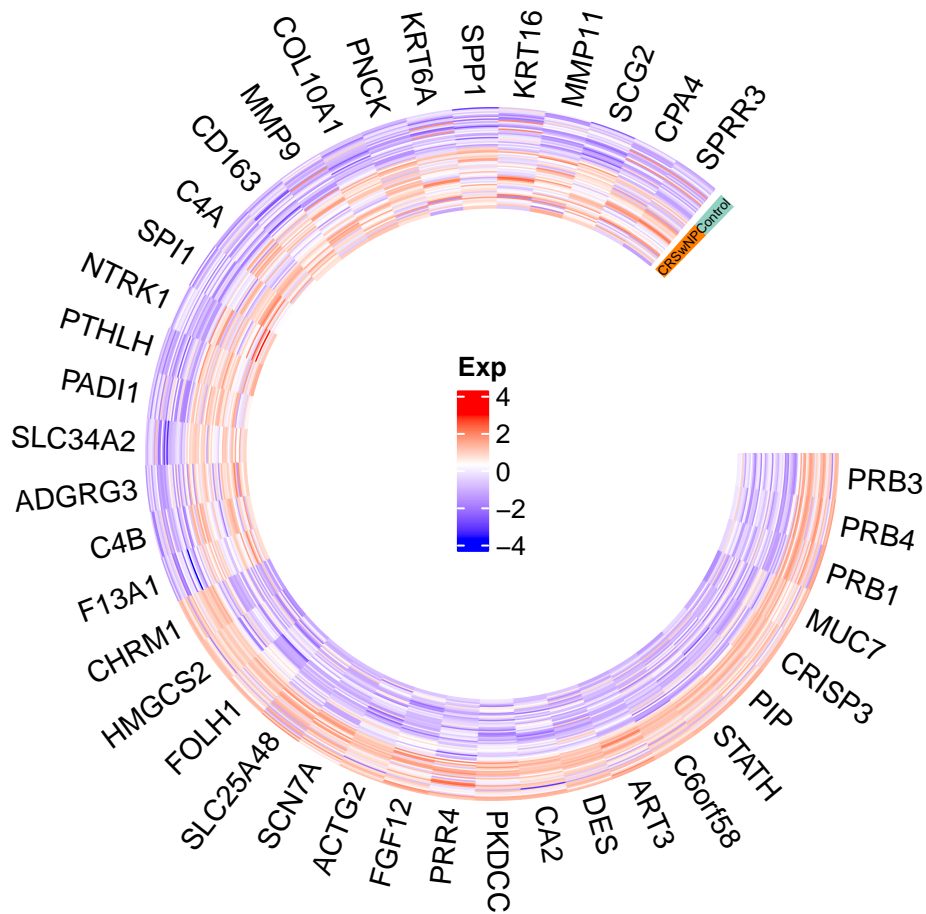

Supplement: Supplementary file 1 [file DataSheet3.ZIP › 原始数据-上传frontiers in genetics/02_result/01_DEG/fig1-2.DEG_circpheatmap.pdf]

DEGs

MRGs

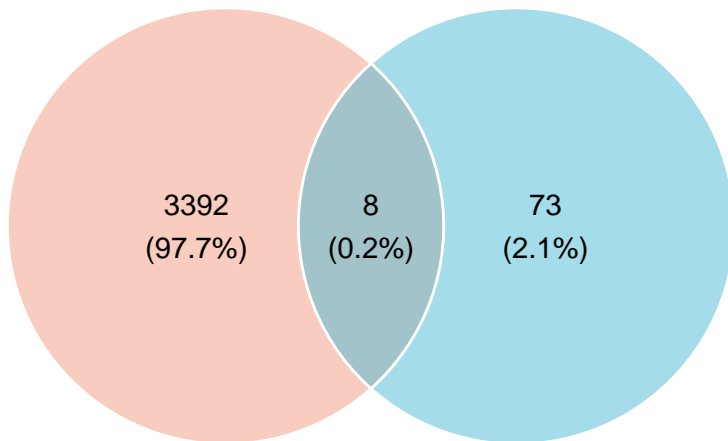

Supplement: Supplementary file 1 [file DataSheet3.ZIP › 原始数据-上传frontiers in genetics/02_result/02_Venn/fig2-1.venn.pdf]

# GO-BP

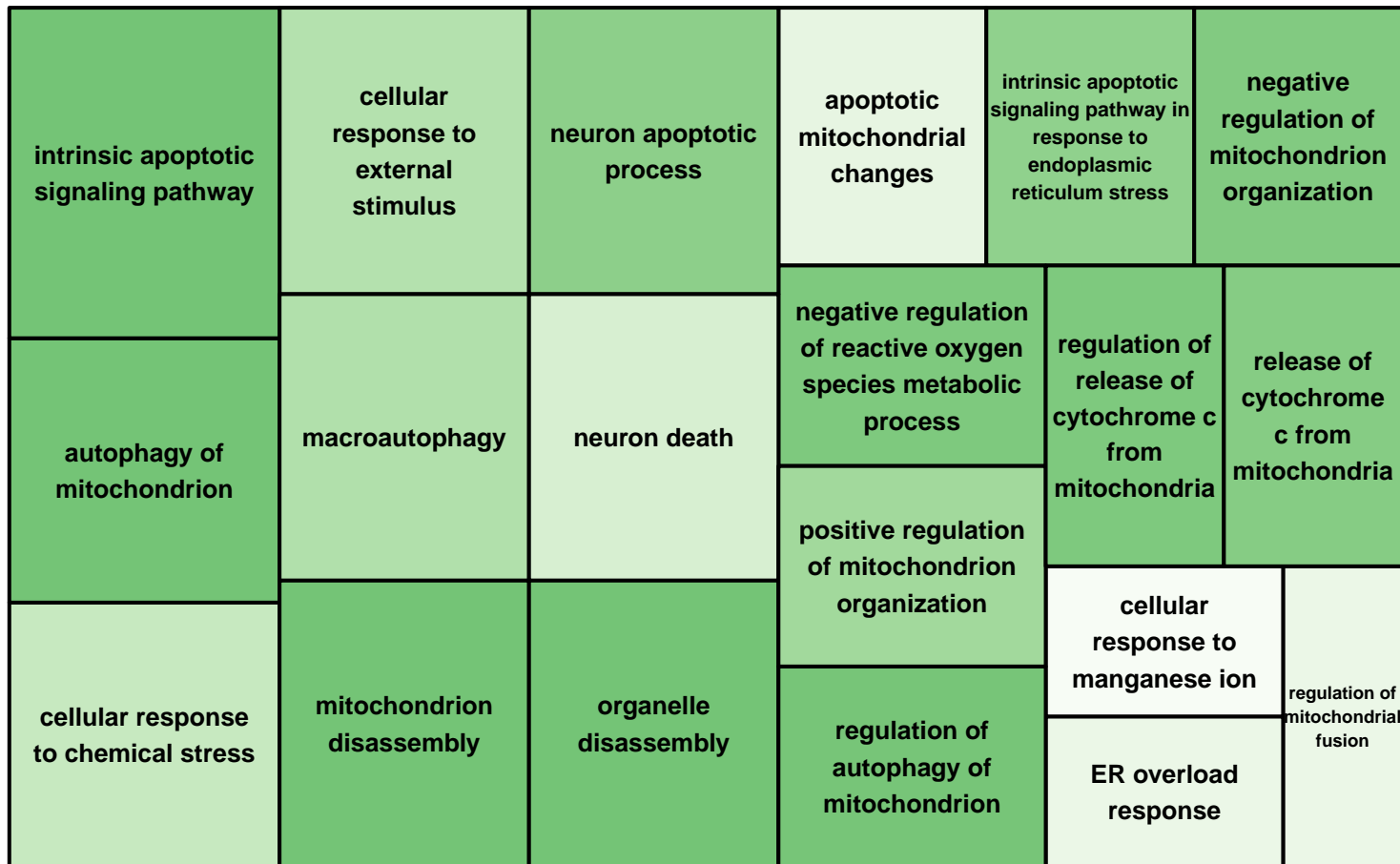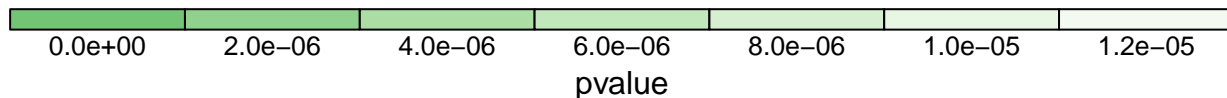

Supplement: Supplementary file 1 [file DataSheet3.ZIP › 原始数据-上传frontiers in genetics/02_result/02_Venn/fig2-2A.GO_BP_treemap_plot.pdf]

# GO-MF

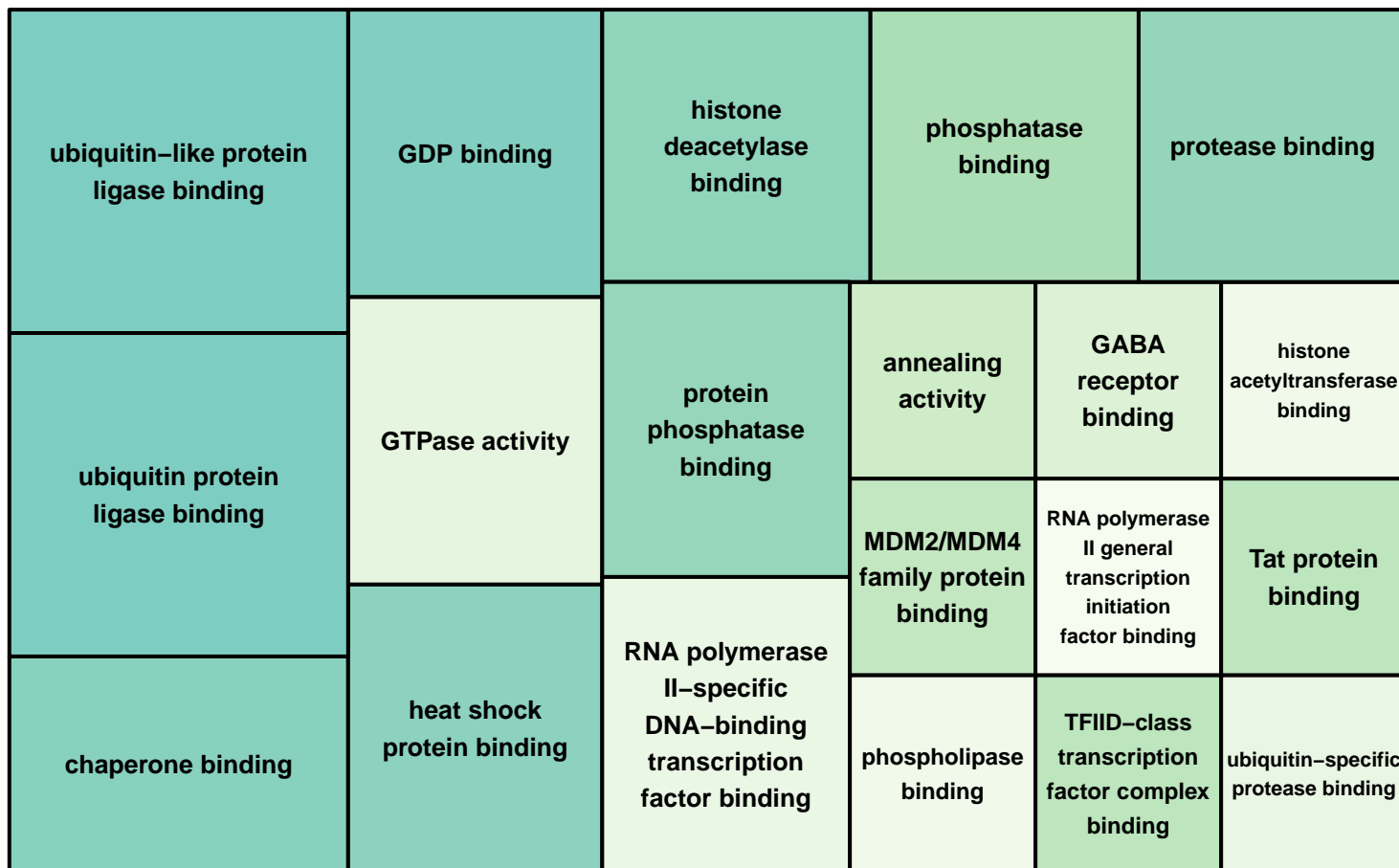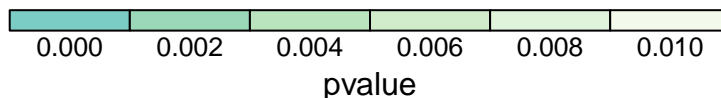

Supplement: Supplementary file 1 [file DataSheet3.ZIP › 原始数据-上传frontiers in genetics/02_result/02_Venn/fig2-2C.GO_MF_treemap_plot.pdf]

# KEGG

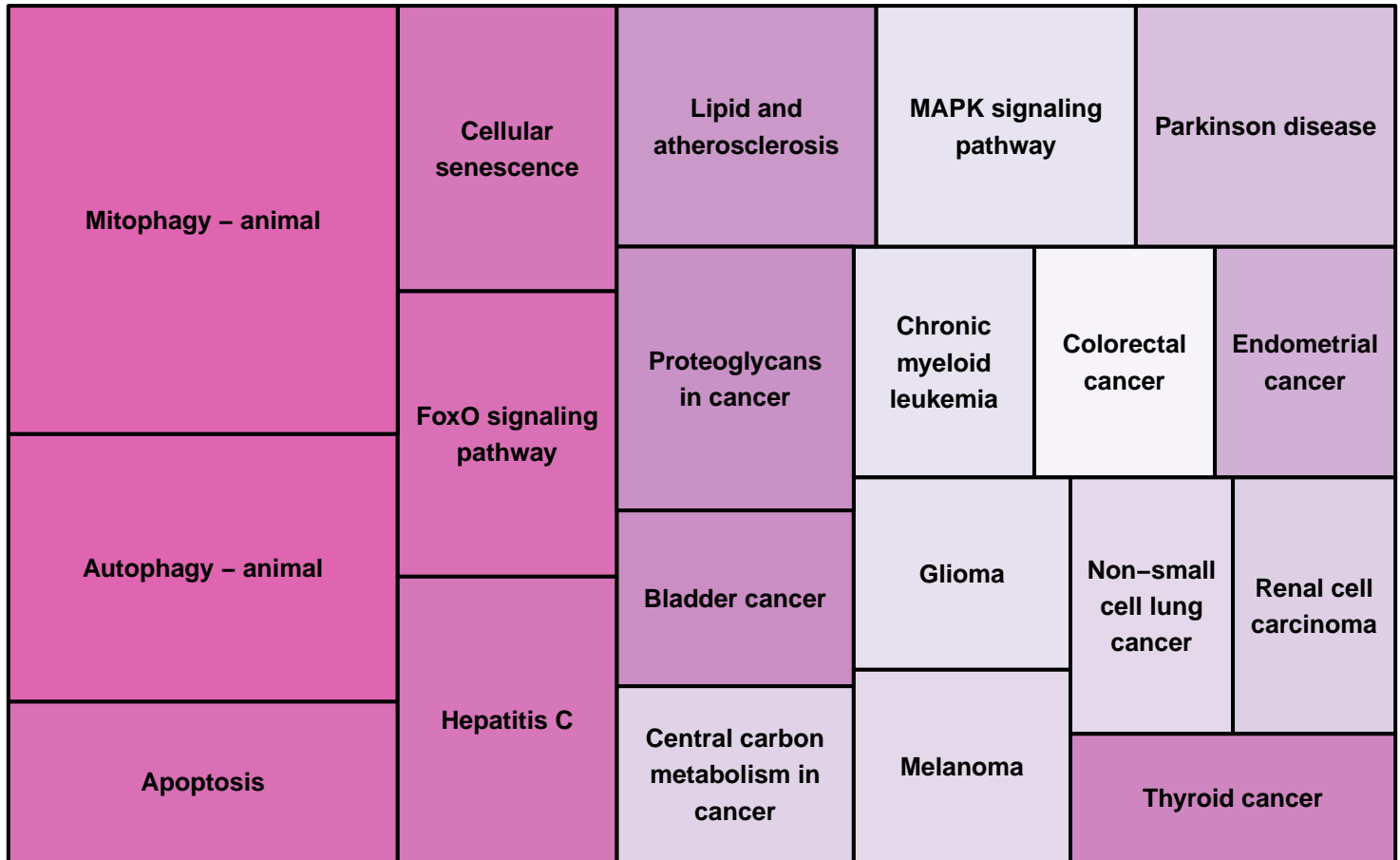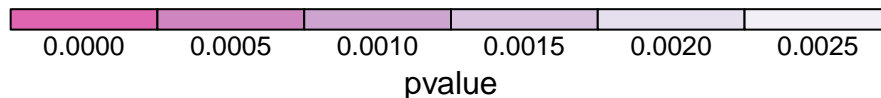

Supplement: Supplementary file 1 [file DataSheet3.ZIP › 原始数据-上传frontiers in genetics/02_result/02_Venn/fig2-3.KEGG_treemap_plot.pdf]

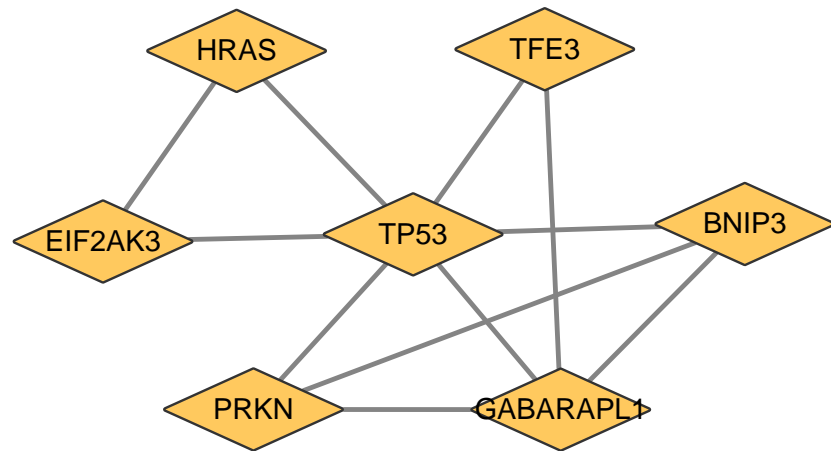

Supplement: Supplementary file 1 [file DataSheet3.ZIP › 原始数据-上传frontiers in genetics/02_result/02_Venn/fig2-4.PPI.pdf]

Binomial Deviance

1.4  
1.2  
1.0  
0.8

8 8 8 8 8 8 8 8 8 7 7 6 6 5 5 5 5 3

-8 -7 -6 -5 -4 -3 -2

Log Lambda

Lambda.min  
0.021

Lambda.lse  
0.086

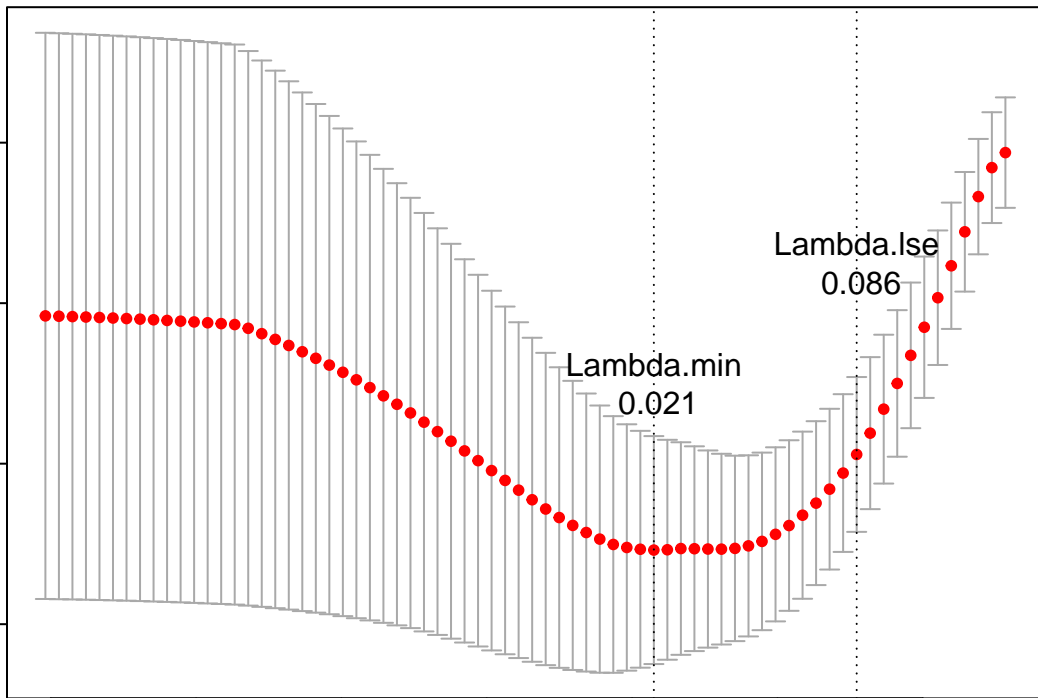

Supplement: Supplementary file 1 [file DataSheet3.ZIP › 原始数据-上传frontiers in genetics/02_result/03_Machine/fig3-1A.lasso.Binomial.Deviance.pdf]

Coefficients

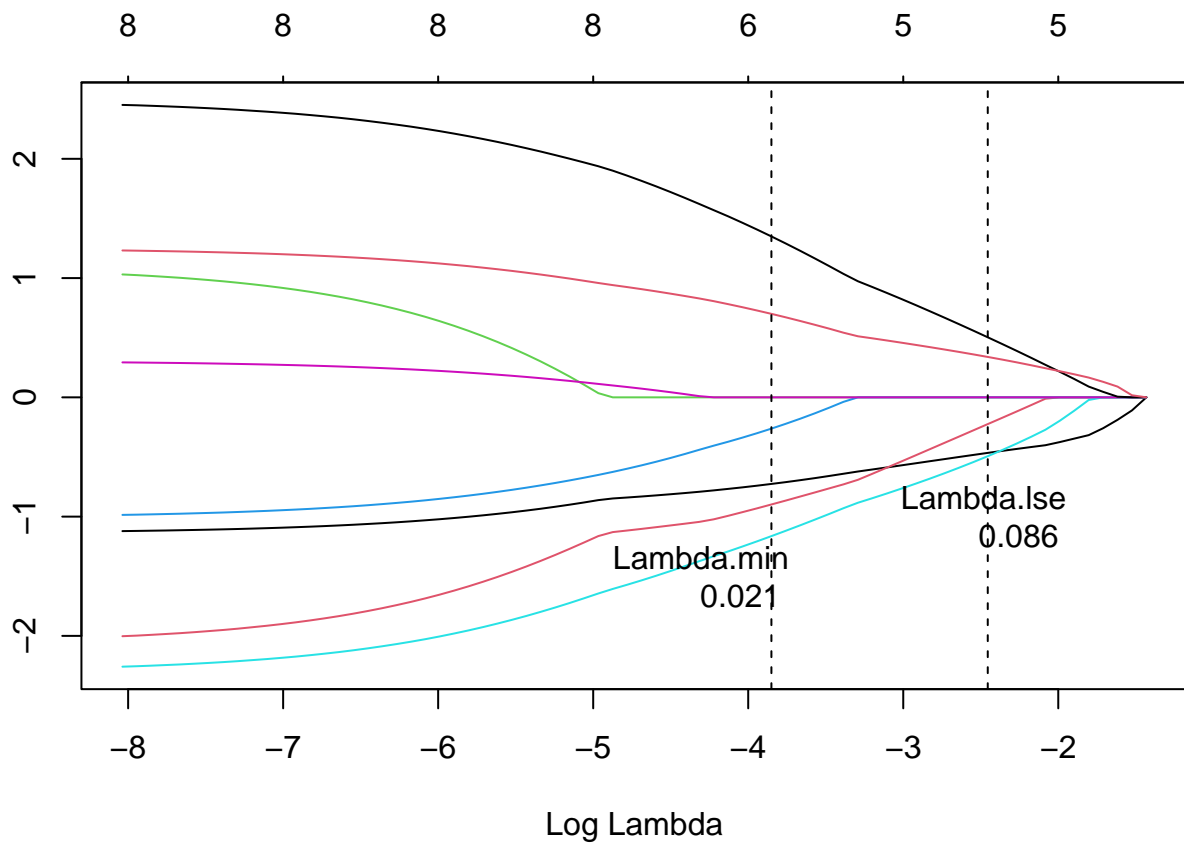

Supplement: Supplementary file 1 [file DataSheet3.ZIP › 原始数据-上传frontiers in genetics/02_result/03_Machine/fig3-1B.lasso.voefficients.venalty.pdf]

Importance

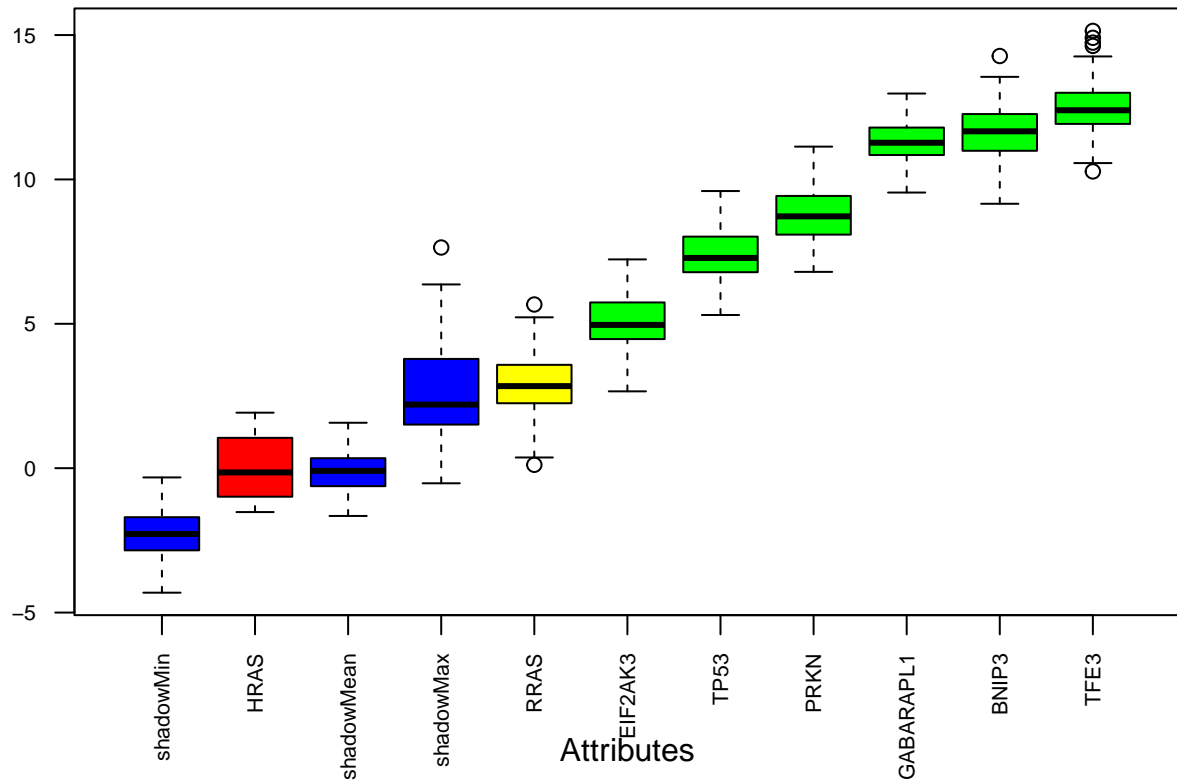

Supplement: Supplementary file 1 [file DataSheet3.ZIP › 原始数据-上传frontiers in genetics/02_result/03_Machine/fig3-2.Boruta.pdf]

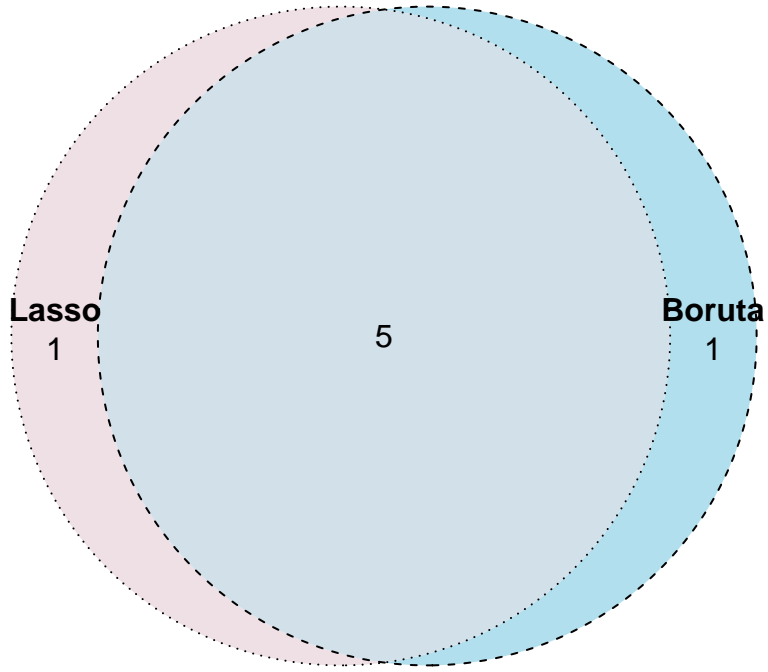

Supplement: Supplementary file 1 [file DataSheet3.ZIP › 原始数据-上传frontiers in genetics/02_result/03_Machine/fig3-3.machine_venn_gene.pdf]

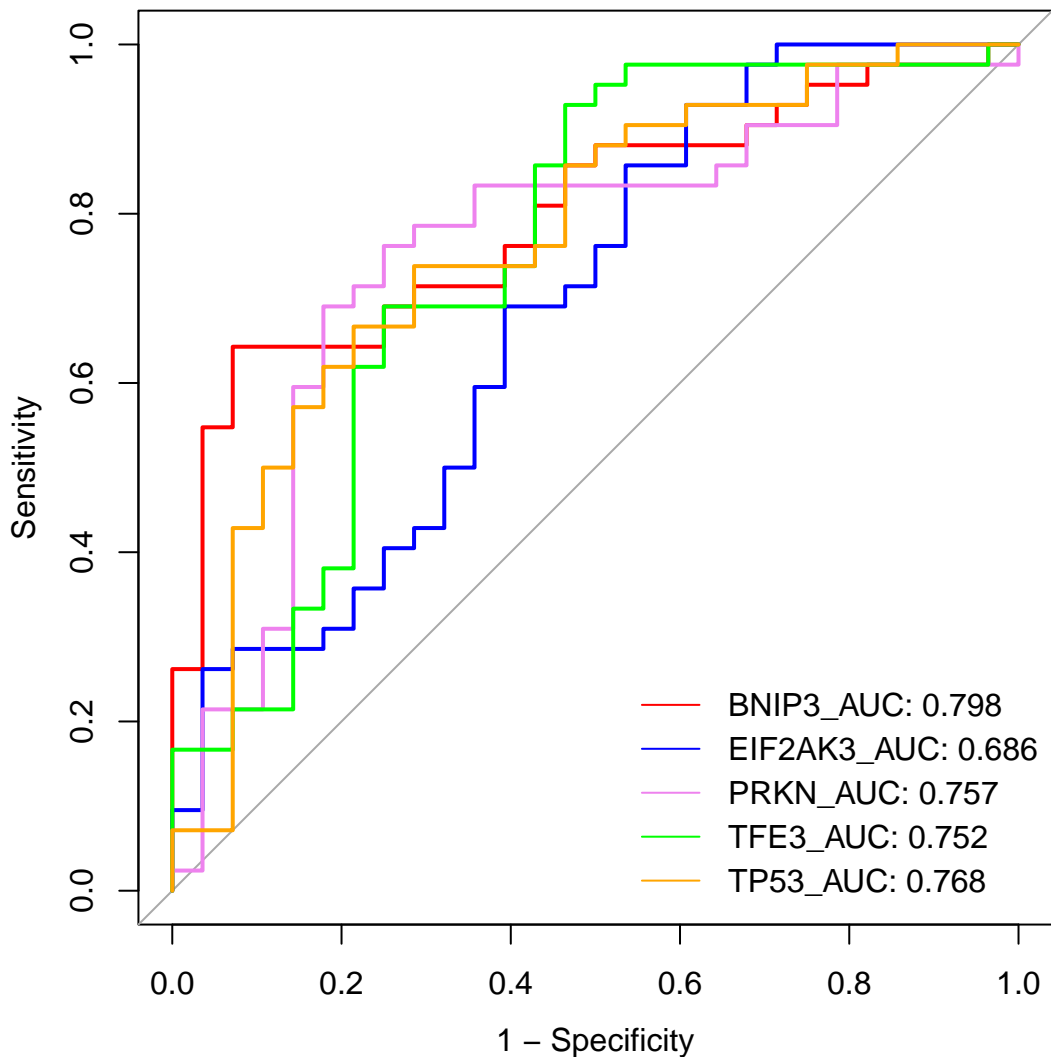

Supplement: Supplementary file 1 [file DataSheet3.ZIP › 原始数据-上传frontiers in genetics/02_result/03_Machine/fig3-4A.GSE136825_gene_ROC.pdf]

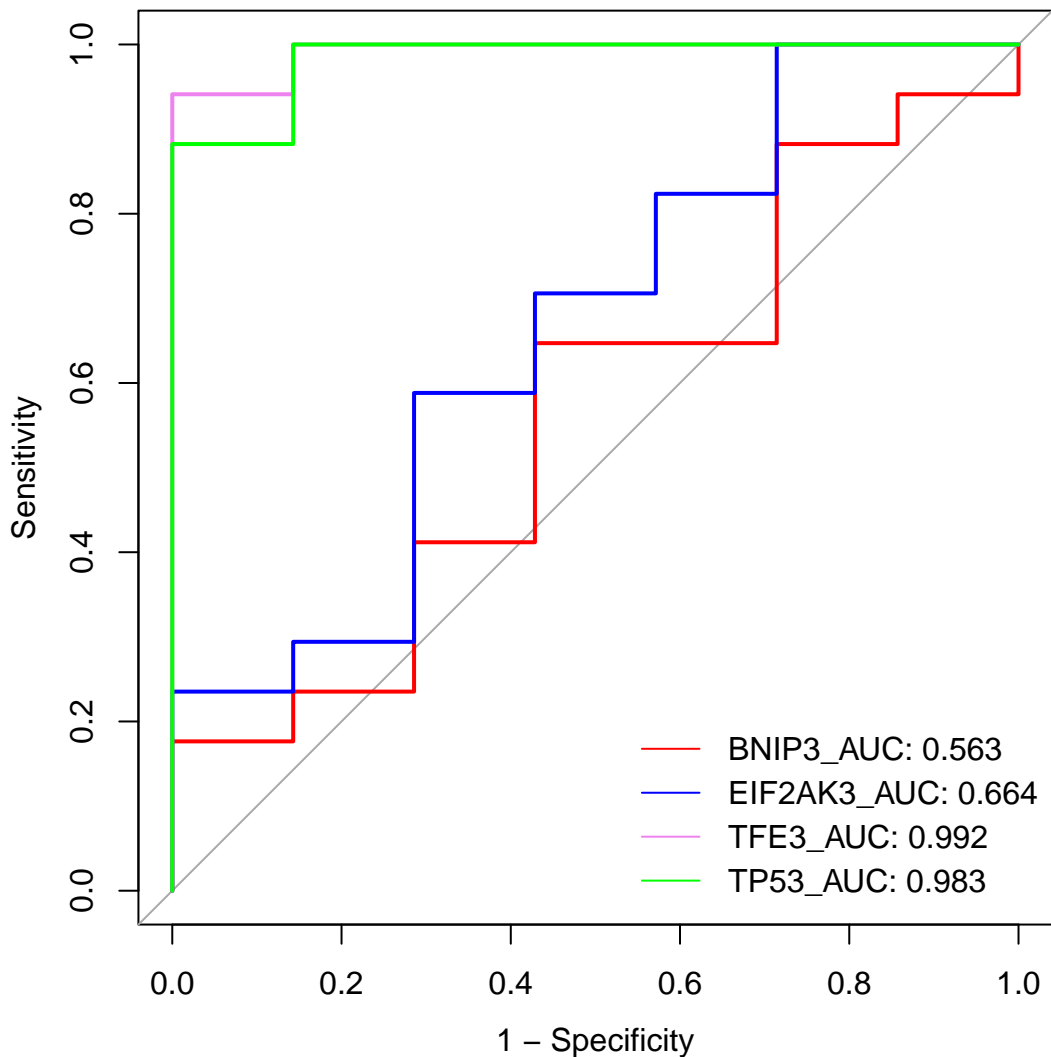

Supplement: Supplementary file 1 [file DataSheet3.ZIP › 原始数据-上传frontiers in genetics/02_result/03_Machine/fig3-4B.testGSE179265_gene_ROC.pdf]

# GSE136825

Group 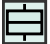 Control 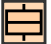 CRSwNP

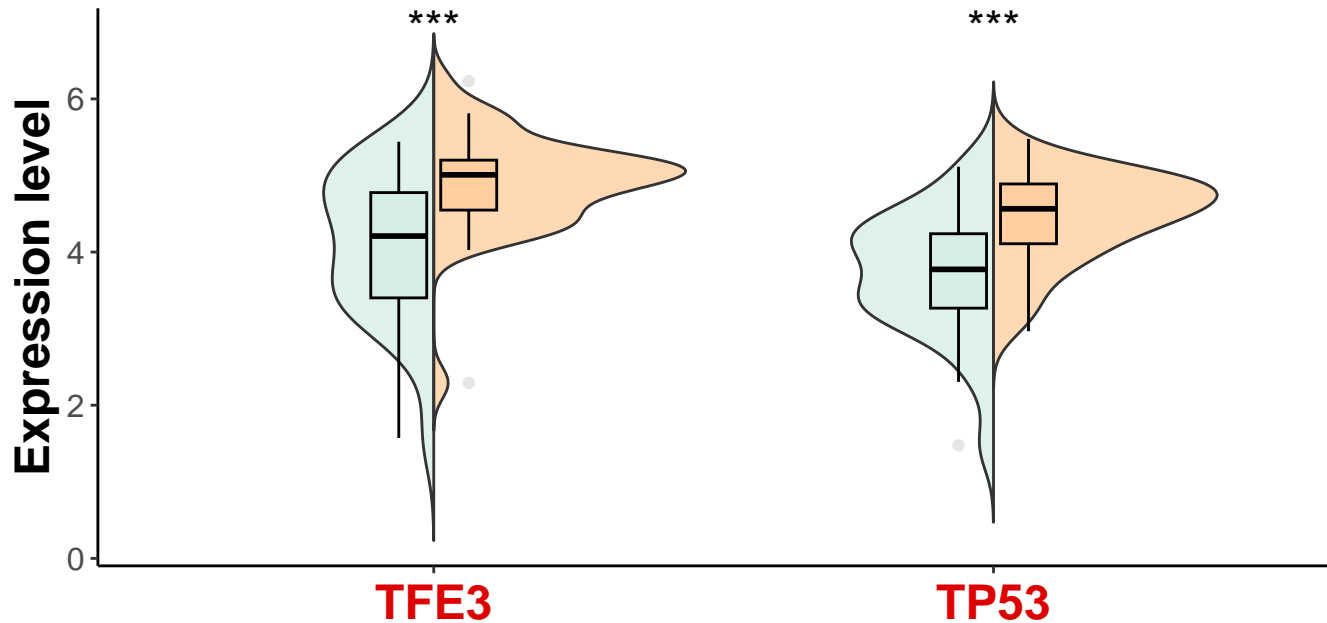

Supplement: Supplementary file 1 [file DataSheet3.ZIP › 原始数据-上传frontiers in genetics/02_result/03_Machine/fig3-5A.train_GSE136825_Expr.pdf]

# GSE179265

Group 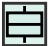 Control 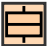 CRSwNP

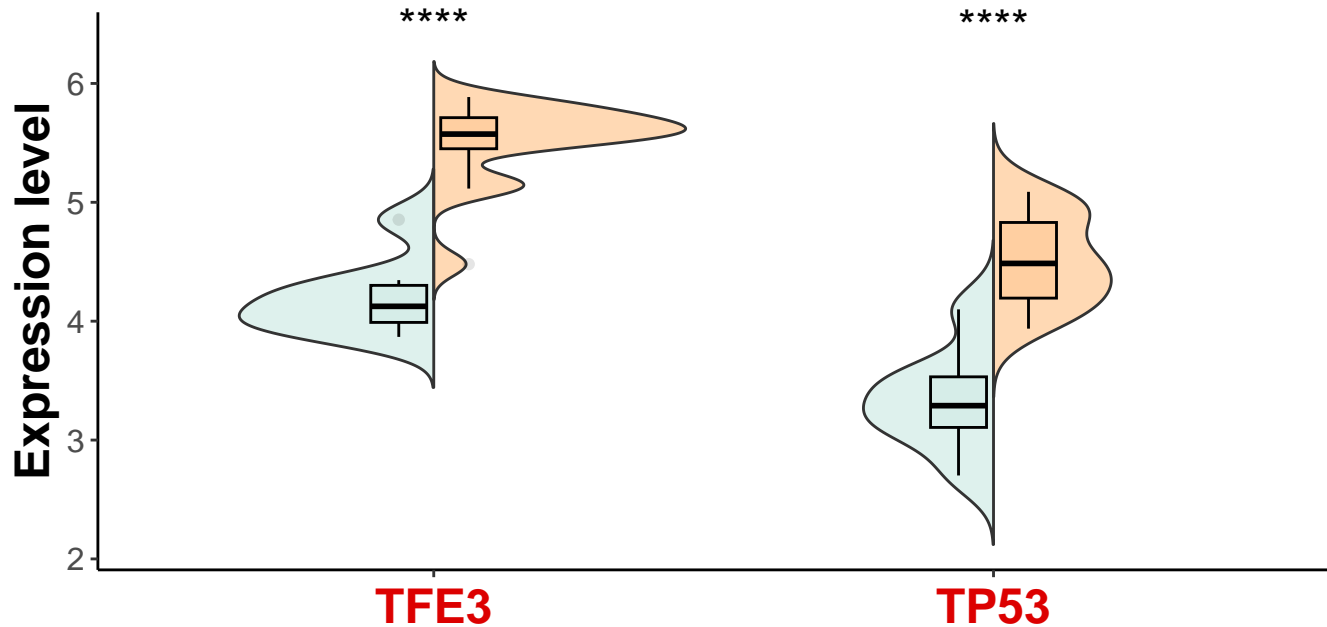

Supplement: Supplementary file 1 [file DataSheet3.ZIP › 原始数据-上传frontiers in genetics/02_result/03_Machine/fig3-5B.test_GSE179265_Expr.pdf]

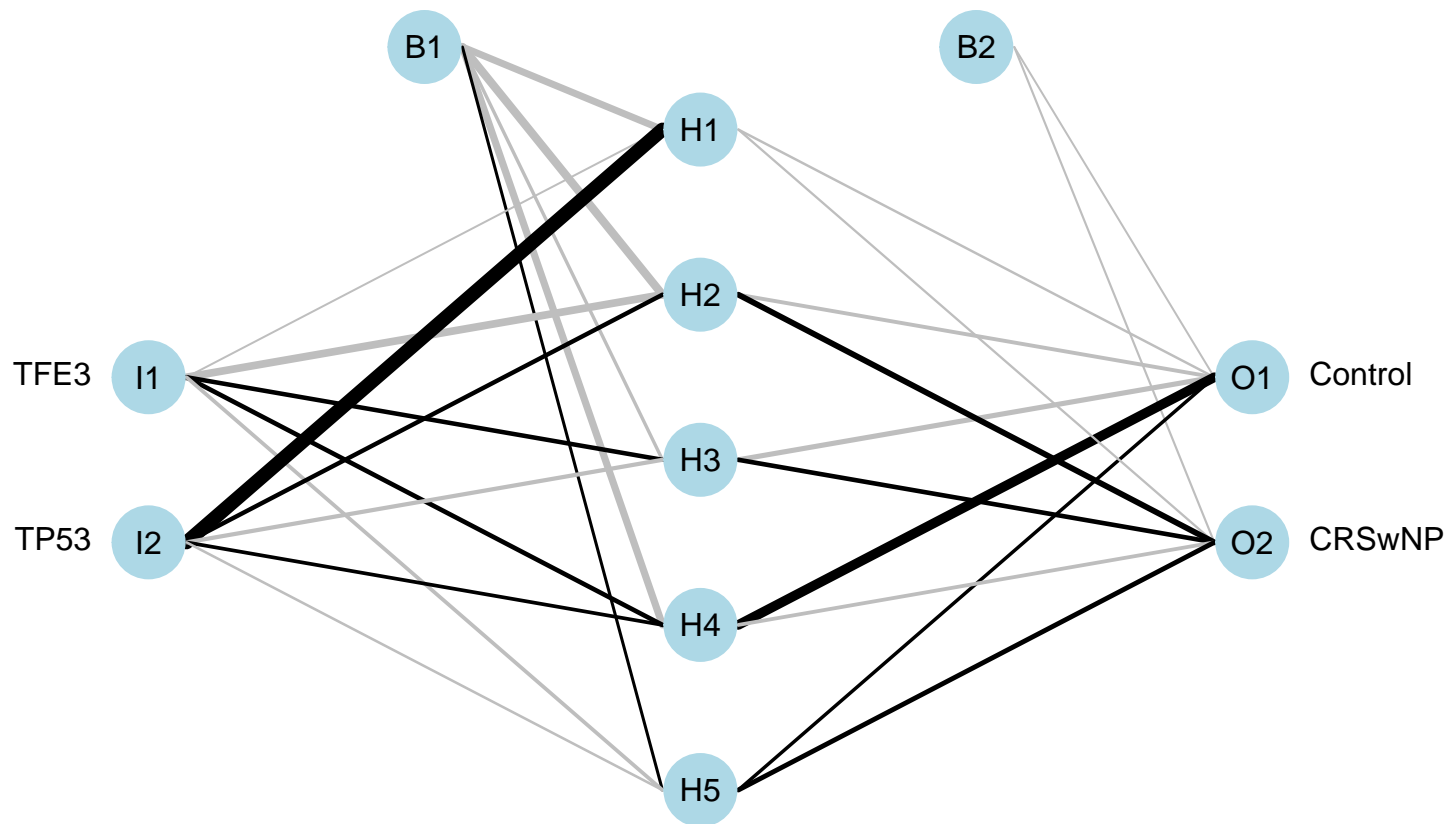

Supplement: Supplementary file 1 [file DataSheet3.ZIP › 原始数据-上传frontiers in genetics/02_result/04_BP/fig4-1.neuralnet_BP.pdf]

**Train group**

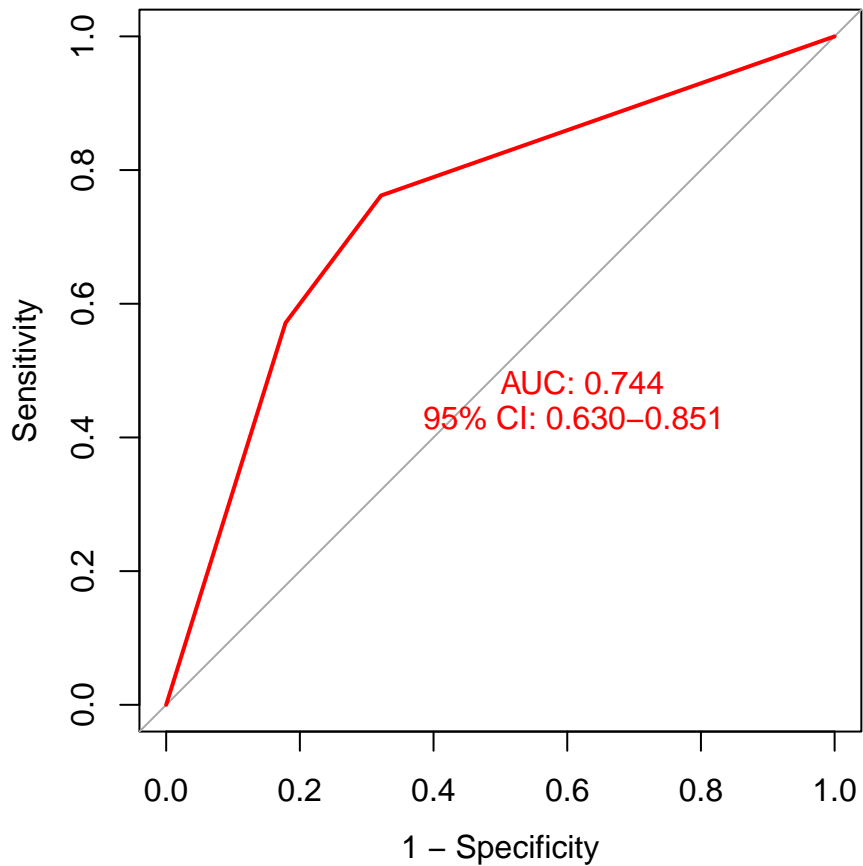

Supplement: Supplementary file 1 [file DataSheet3.ZIP › 原始数据-上传frontiers in genetics/02_result/04_BP/fig4-2.ROC.pdf]

TP53

$R = 0.7, p = 1.3e-11$

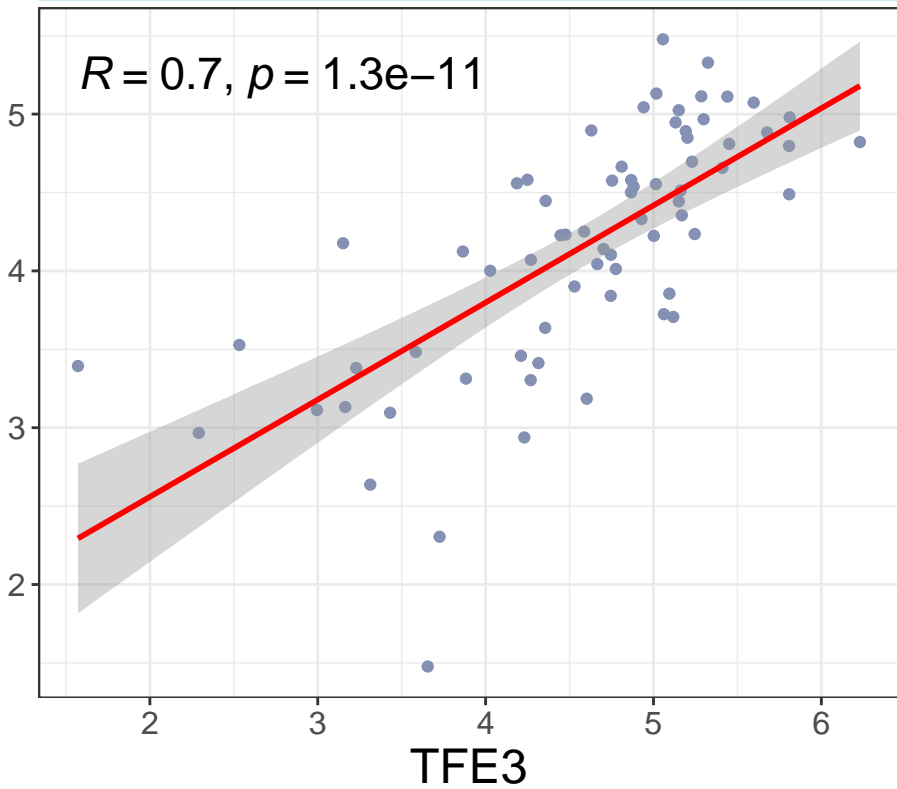

Supplement: Supplementary file 1 [file DataSheet3.ZIP › 原始数据-上传frontiers in genetics/02_result/05_Cor/fig5-1.cor_hub.pdf]

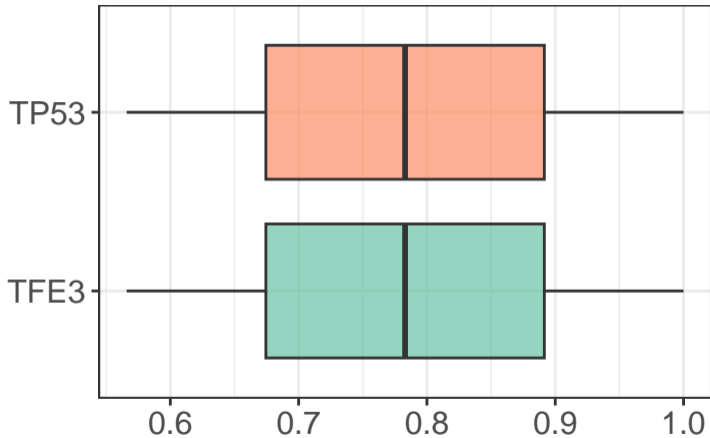

Supplement: Supplementary file 1 [file DataSheet3.ZIP › 原始数据-上传frontiers in genetics/02_result/05_Cor/fig5-2.Gene_function_boxplot.pdf]

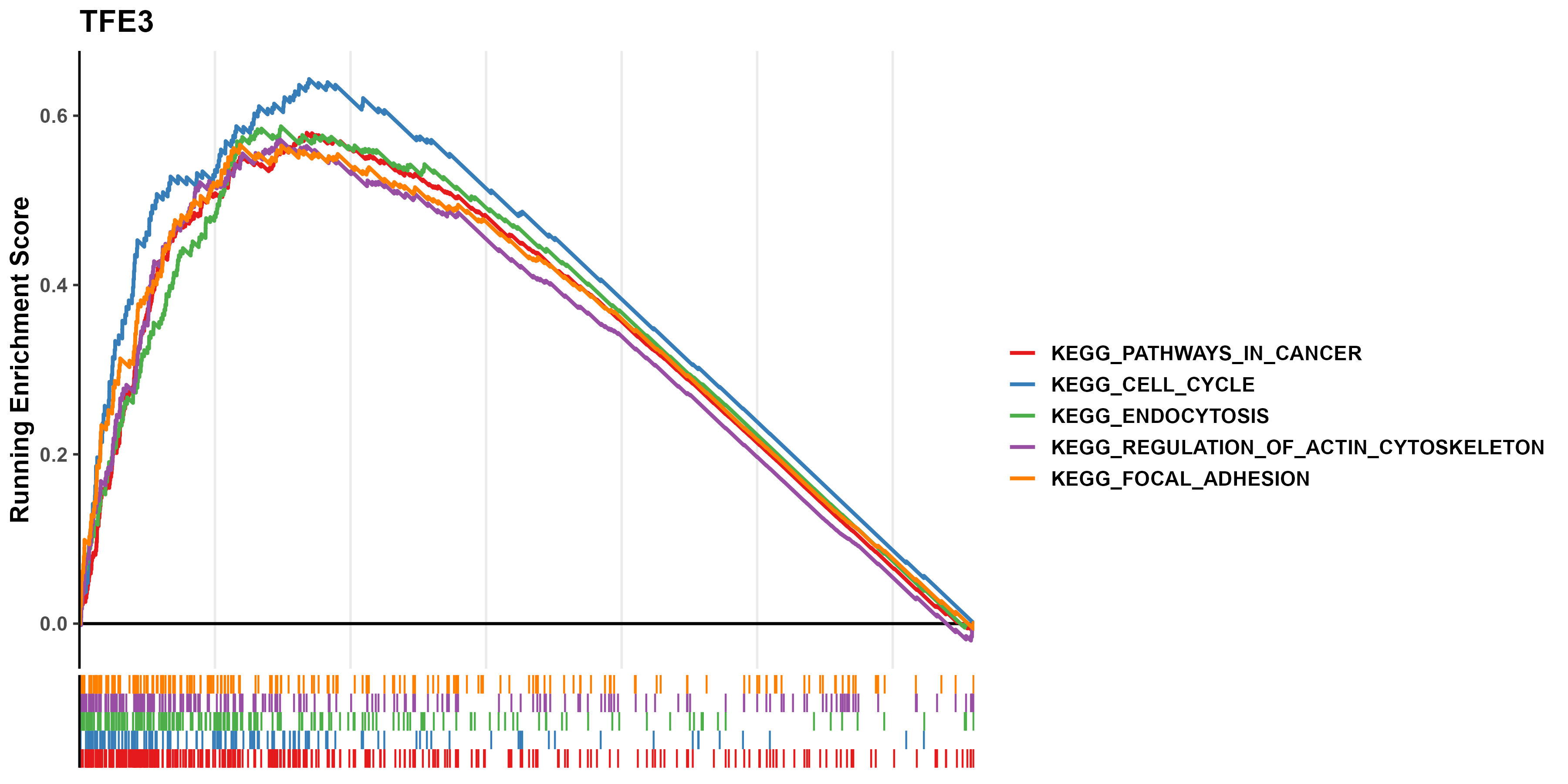

Supplement: Supplementary file 1 [file DataSheet3.ZIP › 原始数据-上传frontiers in genetics/02_result/06_GSEA/02.GSEA/01.TFE3.png]

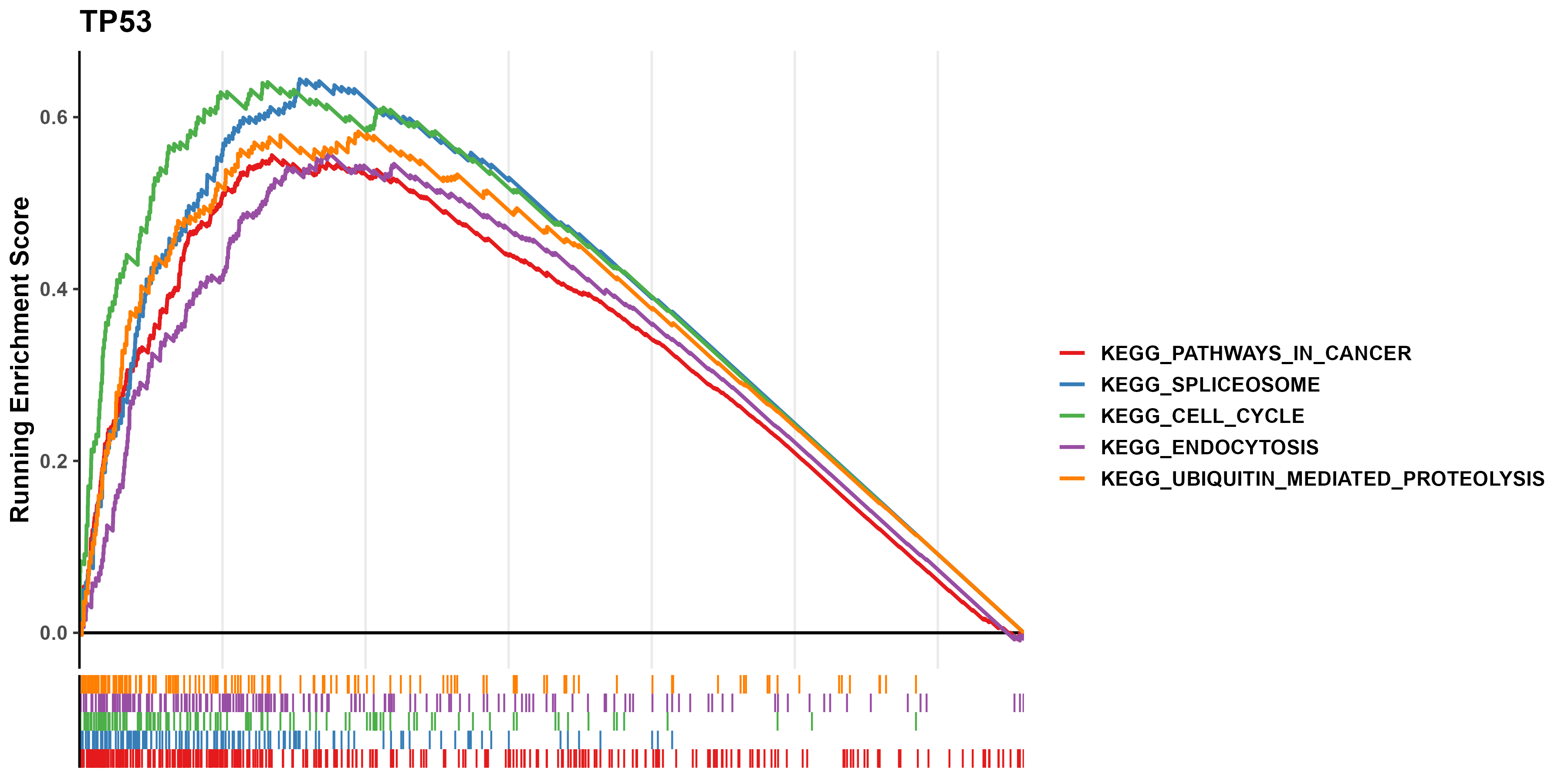

Supplement: Supplementary file 1 [file DataSheet3.ZIP › 原始数据-上传frontiers in genetics/02_result/06_GSEA/02.GSEA/02.TP53.png]

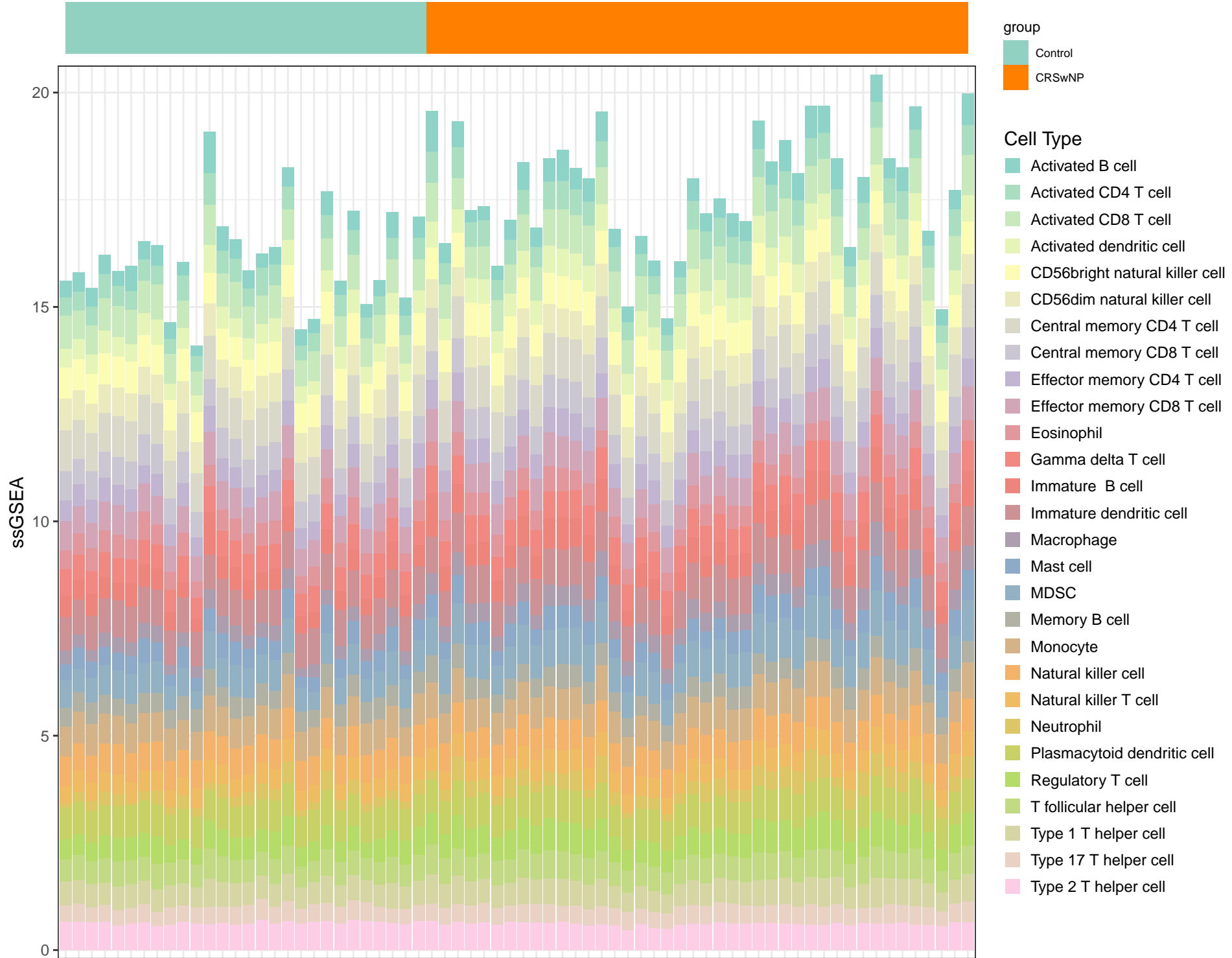

Supplement: Supplementary file 1 [file DataSheet3.ZIP › 原始数据-上传frontiers in genetics/02_result/07_Immune/fig7-1A.Immune_Cell_rate.pdf]

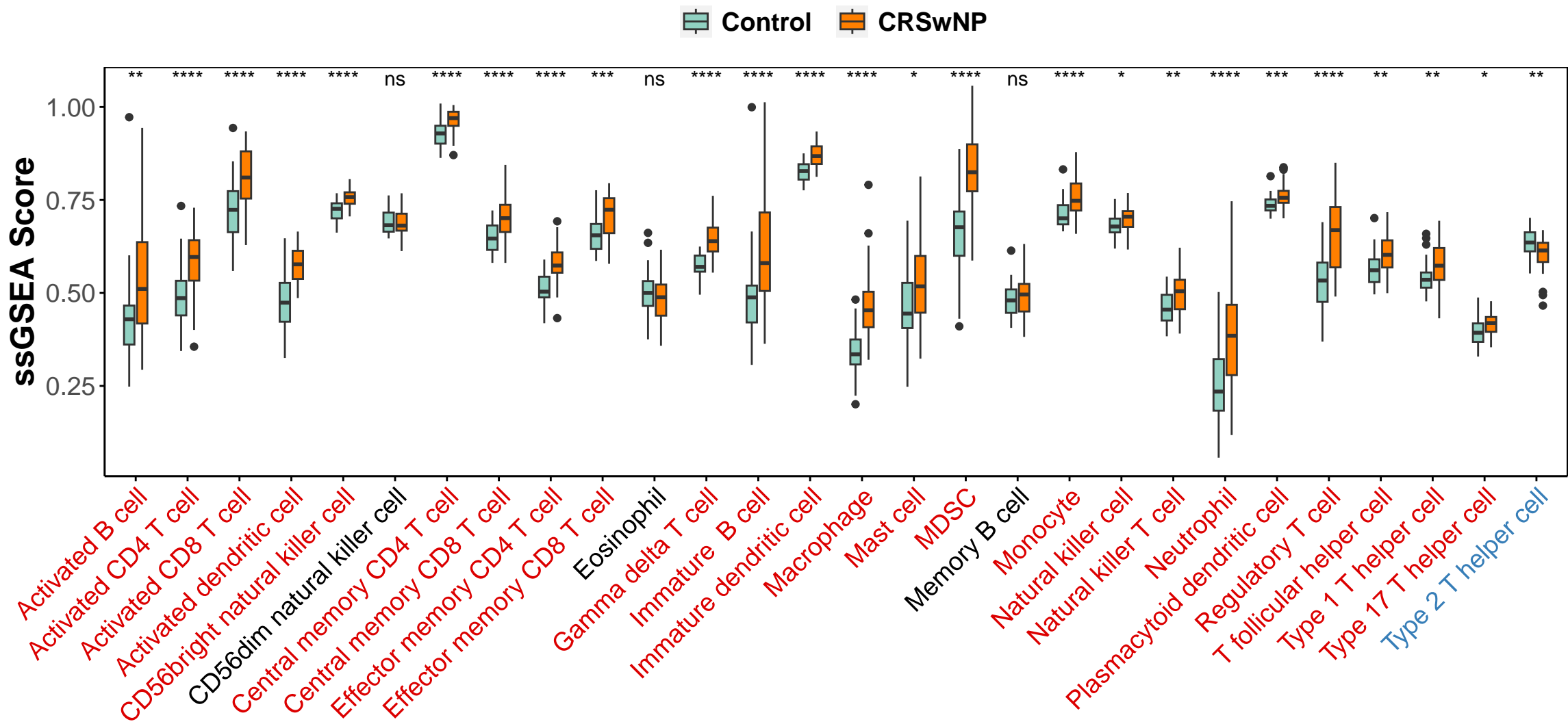

Supplement: Supplementary file 1 [file DataSheet3.ZIP › 原始数据-上传frontiers in genetics/02_result/07_Immune/fig7-1B.Immune_Box.pdf]

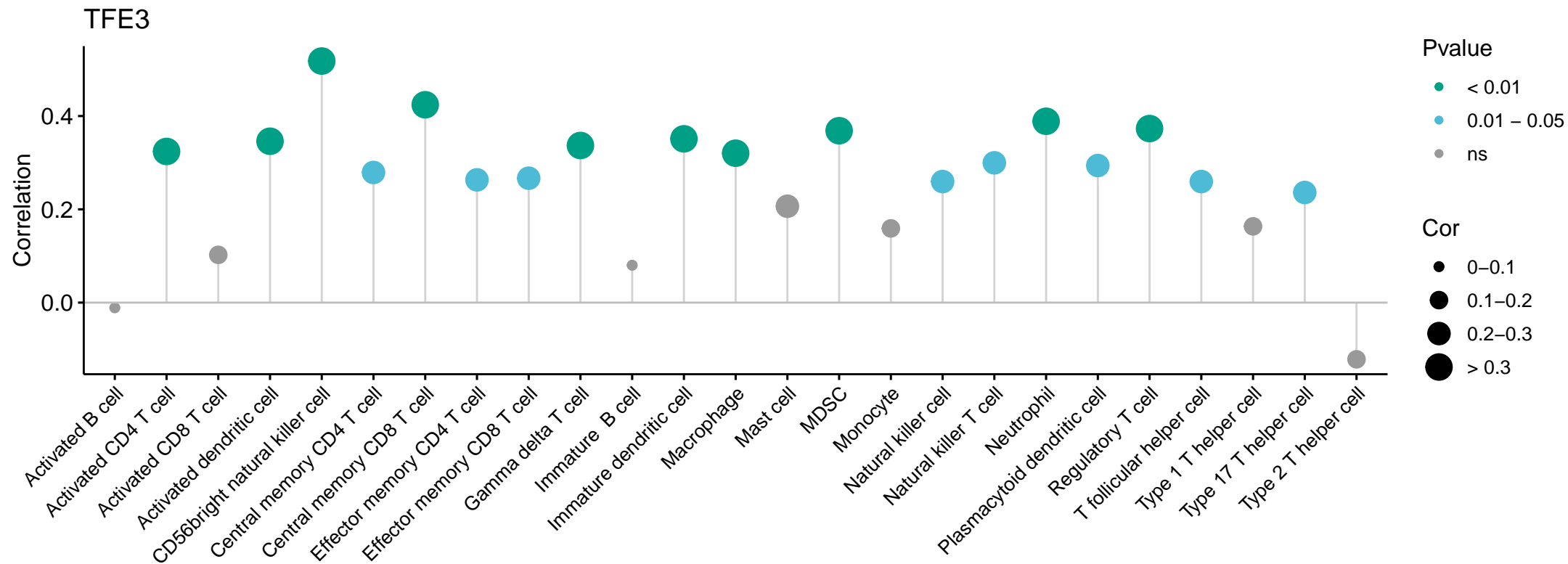

Supplement: Supplementary file 1 [file DataSheet3.ZIP › 原始数据-上传frontiers in genetics/02_result/07_Immune/fig7-2A.TFE3_DEImmune_cor.pdf]

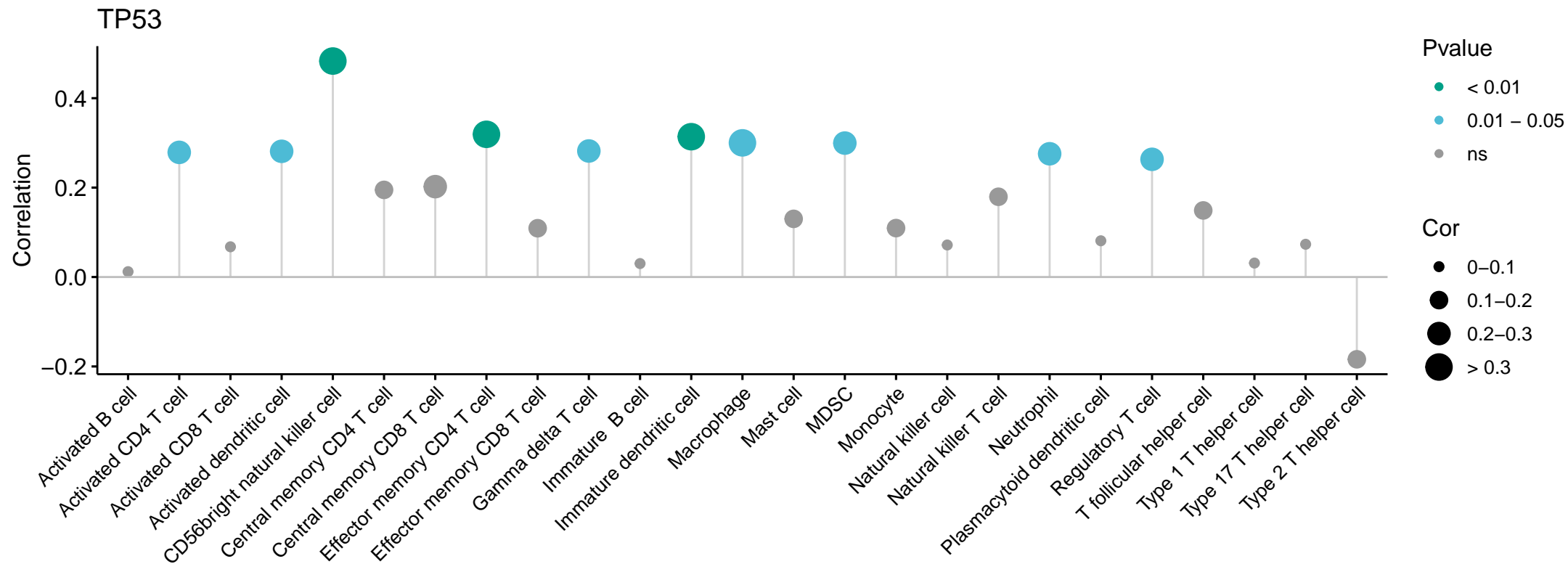

Supplement: Supplementary file 1 [file DataSheet3.ZIP › 原始数据-上传frontiers in genetics/02_result/07_Immune/fig7-2B.TP53_DEImmune_cor.pdf]

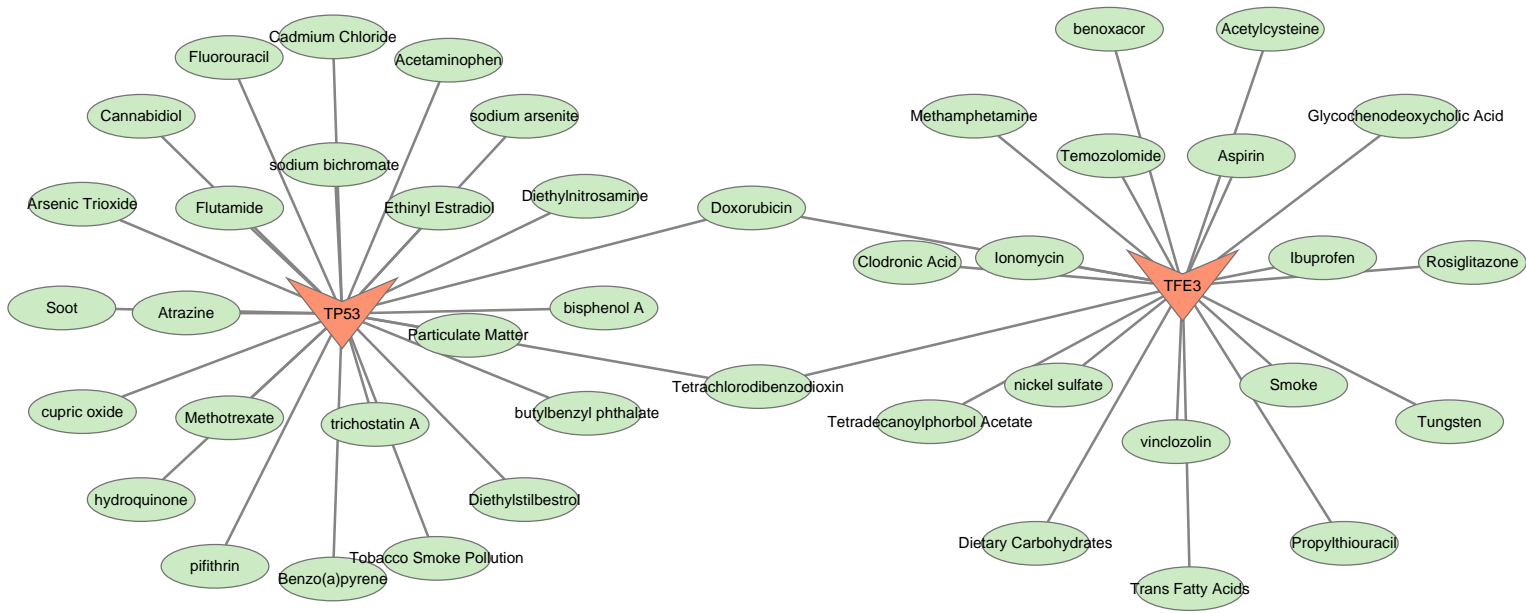

Supplement: Supplementary file 1 [file DataSheet3.ZIP › 原始数据-上传frontiers in genetics/02_result/10_Drug/fig10.Drug.pdf]

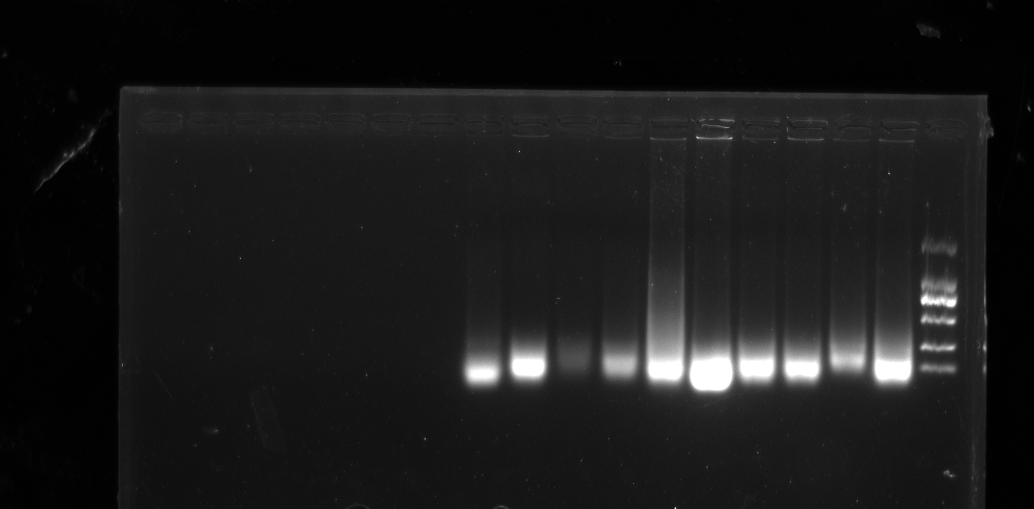

Supplement: Supplementary file 2 [file DataSheet1.ZIP › 5.21上传-PCR凝胶原图/231115-9_8bit.tif]
